# The Genus *Adonis* as an Important Cardiac Folk Medicine: A Review of the Ethnobotany, Phytochemistry and Pharmacology

**DOI:** 10.3389/fphar.2019.00025

**Published:** 2019-02-04

**Authors:** Xiaofei Shang, Xiaolou Miao, Feng Yang, Chunmei Wang, Bing Li, Weiwei Wang, Hu Pan, Xiao Guo, Yu Zhang, Jiyu Zhang

**Affiliations:** ^1^Key Laboratory of New Animal Drug Project, Lanzhou Institute of Husbandry and Pharmaceutical Sciences, Chinese Academy of Agricultural Sciences, Lanzhou, China; ^2^Key Laboratory of Veterinary Pharmaceutical Development of Ministry of Agriculture, Lanzhou Institute of Husbandry and Pharmaceutical Sciences, Chinese Academy of Agricultural Sciences, Lanzhou, China; ^3^Tibetan Medicine Research Center, Qinghai University Medical College, Qinghai University, Xining, China; ^4^PLA Lanzhou General Hospital, Lanzhou, China

**Keywords:** *Adonis* L., cardiac glycosides, cardiovascular activity, toxicity, resources

## Abstract

The genus *Adonis* L. (Ranunculaceae), native to Europe and Asia, comprises 32 annual or perennial herbaceous species. Due to their cardiac-enhancing effects, *Adonis* spp. have long been used in European and Chinese folk medicine. These plants have been widely investigated since the late 19th century, when the cardiovascular activity of *Adonis vernalis* L. was noted in Europe. The present paper provides a review of the phytochemistry, biological activities and toxicology in order to highlight the future prospects of the genus. More than 120 chemical compounds have been isolated, with the most important components being cardiac glycosides as well as flavones, carotenoids, coumarins and other structural types. Plants of the genus, especially *A. vernalis* L. and *A. amurensis* Regel & Radde, their extracts and their active constituents possess broad pharmacological properties, including cardiovascular, antiangiogenic, antibacterial, antioxidant, anti-inflammatory and acaricidal activities, and exhibit both diuretic effects and effects on the central nervous system. However, most plants within the 32 species have not been comprehensively studied, and further clinical evaluation of their cardiovascular activity and toxicity should be conducted after addressing the problem of the rapidly decreasing resources. This review provides new insight into the genus and lays a solid foundation for further development of *Adonis*.

## Introduction

The genus *Adonis* L. (Ranunculaceae), native to Europe and Asia, comprises 32 annual or perennial herbaceous species and grows in temperate regions of the northern hemisphere (Ghorbani et al., 2008; Orhan et al., 2017). The genus was named after the Greek mythological character, and *Adonis* spp. have long been used in European and Chinese folk medicine for their cardiac-enhancing effects (Abduchamidov et al., 1971; Felter and Lloyd, 2006). Due to the marked effects on heart disease, researchers began focusing attention on the genus *Adonis* (Shikov et al., 2014). With advancements in phytochemistry research, greater numbers of compounds were isolated from the plants of this genus (Heyl et al., 1918); the compounds exhibiting significant cardiovascular activity were primarily classified as cardiac glycosides (Katz and Reichstein, 1947; Deng et al., 1963; Chi et al., 1985). These reports further substantiated the traditional uses of these plants for cardiac enhancement (Shikov et al., 2014). Moreover, flavones, carotenoids, coumarins and other structural classes were identified, and additional pharmacological activities were found, including antiangiogenic, antibacterial, antioxidant and anti-inflammatory activities, as well as effects on the central nervous system, a diuretic effect and acaricidal activity (May and Willuhn, 1978; Gu et al., 1980; Wang et al., 1981; You et al., 2003; Das et al., 2007; Shang et al., 2012, 2013, 2017; Mohadjerani et al., 2014). These newly discovered compounds and their previously unknown bioactivities advanced and promoted the development of the genus *Adonis* (Yang et al., 2015).

In the late 19th century, the cardiovascular activity of *Adonis vernalis* L. distributed in the Eurasian region was observed. And since the early 20th century, extracts of this plant enriched in cardiac glycosides were prepared to treat chronic heart failure in the former Soviet Union and Germany. In China and other East Asian countries including Korea, and Japan, *A. amurensis* Regel & Radde was studied and used to treat heart diseases in the mid-20th century due to a shortage of cardiotonic agents (Deng et al., 1963). Additionally, the toxicity of these plants became apparent, and *Adonis*-induced poisoning cases in both humans and animals were observed (Hurst, 1942; Galey et al., 1996; Woods et al., 2004).

Until recently, researchers have made great advances in studying the phytochemical and pharmacological activities of genus *Adonis*. However, no review article discussing these achievements is available in the literature. This review strives for a complete overview of the existing botanical knowledge, traditional uses, phytochemistry and pharmacological research of species belonging to the genus *Adonis*. Available information on these species enables us to explore their therapeutic potential, to highlight the gaps in our knowledge and to provide the scientific basis for future research.

## Methods

As well as two reviews published by Kooti et al. (2016, 2018), in this review we searched the information on this genus from databases (using Elsevier, ACS, Springer, Wiley, Nature, RSC, Medline Plus, Bentham Science, Hindawi Science, CNKI, VIP, Web of Science, Google Scholar and Baidu Scholar) and libraries, and the search languages were set to English and Chinese. We didn’t set the time period for searching more literatures. The keywords were searched as *Adonis* for English literatures, Cejinzhan (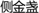) and/or Fushoucao (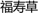) for Chinese literatures. Three experts collected the literatures.

## Botany

**FIGURE 1:**
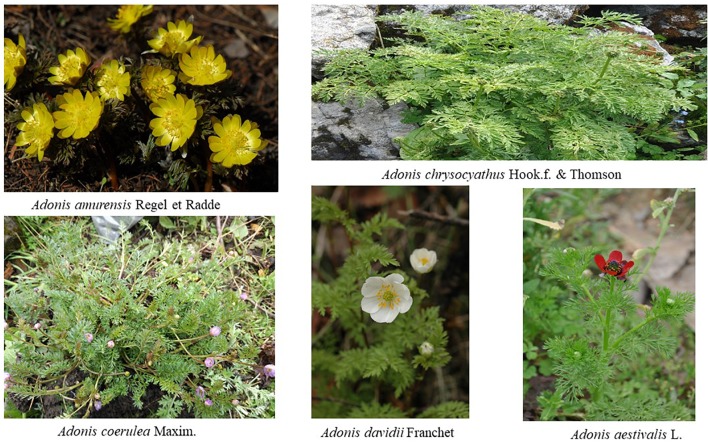
The pictures of five plants grown in China.

The generic name *Adonis* refers to the mythic character Adonis, a lover of the goddess Aphrodite or Venus. Plants belonging to the *Adonis* genus are native to Europe and Asia and have been introduced to North America. It includes approximately 32 annual or perennial herbaceous species of flowering plants of Ranunculaceae. In “The Plant List,” 143 scientific plant names of species rank for the genus *Adonis* are included, and of these 32 are accepted species names (The plant list, 2013). Basal and lower stem leaves are usually scaly and upper stem leaves alternate and are palmately or pinnately divided. One-flowered inflorescences terminate on branches or branchlets with absent bracts. The flowers are radially symmetric, bisexual and usually red, orange, or yellowish, having 5 to 30 petals. The plants possess numerous stamens and spirally arranged pistils, linear filaments, and one-ovuled ovaries with persistent styles and small stigma. The plants have achenes, usually with raised veins, and the leaves and roots are poisonous to humans and livestock (Heyn and Pazy, 1989; Gostin, 2011; Flora of China, 2018). Due to the beauty of the flower, the plants of this genus were used historically for ornamental purposes in some countries. Only in Germany, the former Soviet Union and some East Asian countries some species and their extracts were used as cardiac agents, especially *A. vernalis* and *A. amurensis* (Table 1 and Figure 1).

**Table 1:** The accepted plant names by The Plant List^∗^.

Name	Distribution	Traditional uses	Others	Reference
*Adonis aestivalis* L.	Native to Europe and Asia, was introduced into North America	Medicinal and ornamental plant	Stems 10–20 cm tall. Sepals narrowly rhombic to narrowly ovate, membranous. Petals orange.	Burrows and Tyrl, 2001
*Adonis amurensis* Regel & Radde	Native to Japan, Russia, Korea, and China	Medicinal plant	Stems 5–15 cm tall in flower, to 30 cm tall in fruit. Flowers 2.8–3.5 cm in diameter, sepals pale grayish purple, Petals yellow.	Shimizu et al., 1967; Flora of China, 2018.
*Adonis annua* L.	Native to North Africa, Western Asia, the Mediterranean, Europe	–	It is endangered and listed as a priority species in United Kingdom	Egger, 1965
*Adonis bobroviana* Simonov.	Native to China	–	Stems to 30 cm tall. Flowers 2–4 cm in diameter Sepals pale green tinged with purple, Petals yellow, abaxially tinged with purple.	Flora of China, 2018
*Adonis chrysocyathus* Hook.f. & Thomson	Native to Greek, and cultivated in the botanical gardens of Copenhagen or Gothenburg	–	Heights from 203 to 381 mm. Orange or yellow flowers. Flower color is variable within the species and changes with drying.	Heyn and Pazy, 1989
*Adonis coerulea* Maxim.	Native to China	Treating mange	Stems 3–15 cm tall. Flowers 1–1.8 cm in diameter Sepals obovate-elliptic to ovate, apex rounded. Petals ca. 8, pale purple to pale blue.	Shang et al., 2013; Flora of China, 2018
*Adonis davidii* Franch.	Native to China and Bhutan	–	Stems 10–58 cm tall. Stem leaves with petiole to 7 cm basally on stem, shortly petiolate or sessile toward stem apex; flowers 1.5–2.8 cm in diameter Sepals glabrous, rarely ciliate. Petals white, sometimes tinged with purple.	Flora of China, 2018.
*Adonis flammea* Jacq.	Distributes in the Anatolia, the Levant Central and Southern Europe	–	It is similar to *A. annua* but is more robust with large flowers with narrow and oblong petals, dark scarlet sepals that are attached to the petals.	Catalogue of Life, 2017
*Adonis microcarpa* DC.	Native to western Asia and southern Europe and is introduced in Australia	–	50 cm tall, has finely divided foliage and red flowers with black centers.	Kloot, 1976
*Adonis multiflora* Nishikawa & Koji Ito	Native to Korea, Japan, and Manchuria	Ornamental plant	20–25 cm tall at flowering with up to four yellow flowers per stem.	Lee et al., 2003
*Adonis ramosa* Franch.	Native to Japan, Russia, Korea, and China	–	Stems 4–20 cm tall, 1.2–2 mm in diameter Flowers 2.5–4 cm in diameter Sepals gray-purple. Petals yellow.	Flora of China, 2018
*Adonis shikokuensis* Nishikawa & Koji Ito. Or *Adonis sibirica* (Patrin ex DC.) Ledeb.	Native to Mongolia, Russia; Europe and China	Medicinal uses	Stems ca. 40 cm tall, 3–5 mm in diameter Sepals yellowish green, rounded-ovate. Petals yellow, narrowly obovate.	Flora of China, 2018
*Adonis sutchuenensis* Franch.	Native to China	–	Stems 15–40 cm tall, Flowers 2–4.8 cm in diameter Sepals pale green, usually oblanceolat. Petals yellow,	Flora of China, 2018
*Adonis tianschanica* (Adolf) Lipsch.	Native to Russia and China	–	Stems ca. 30 cm tall. Flowers 3.5–5 cm in diameter Sepals pale purple, slightly shorter than petals.	Flora of China, 2018
*Adonis vernalis* L.	Natively in central Europe and in Asia	Cardiac stimulant and ornamental plant	The flowers appear in springtime, and are up to 80 mm in diameter, with up to 20 bright yellow petals	Heyl et al., 1918


**^∗^The general description of following species hasn’t been done, including Adonis aleppica Boiss, Adonis apennina L., Adonis cyllenea Boiss., Heldr. & Orph., Adonis dentata Delile, Adonis distorta Ten., Adonis eriocalycina Boiss., Adonis globosa C.H.Steinb. ex Rech.f., Adonis × hybrida C.F.Wolff ex Nyman., Adonis leiosepala Butkov., Adonis mongolica Simonov., Adonis nepalensis Simonov., Adonis nepalensis Simonov., Adonis palaestina Boiss., Adonis pyrenaica DC., Adonis turkestanica (Korsh.) Adolf., Adonis villosa Ledeb., Adonis volgensis Steven ex DC., Adonis wolgensis Steven*.*

## Traditional Uses

*Adonis vernalis*, known as the Bird’s eye, Pheasant’s eye or False Hellebore, is a perennial, dry grassland plant species distributed in the Eurasian region along a 4698-km longitudinal transect from Russia to Spain (Hirsch et al., 2015). This species prefers calcium-rich chernozem soils of various types but also grows in meadow chernozems and gray forest soils (Poluyanova and Lyubarskii, 2008). It is listed in the German Homoeopathic Pharmacopoeia (Shikov et al., 2014). Historically, it was used to treat edema by local people of the former Soviet Union. Extracts of the plant were first introduced into medicine as a cardiac stimulant in 1879 by the Russian medical doctor, N. O. Buhnow, and *A. vernalis* has attracted the interest of many people ever since. In 1898, a mixture of this medicine with sodium bromide (or potassium bromide) or codeine was suggested to treat light forms of epilepsy and heart diseases (Bekhterev, 1898; Shikov et al., 2014). Over the intervening years, an ethanolic extract of the aerial parts of *A. vernalis* was prepared as an alternative cardiac agent in the former Soviet Union. The biological activity of this extract was defined as 50–66 frog units or 6.3–8.0 cat units (Chiang and Mi, 1958; Wagler, 2001). Now, in Russia, the aerial part as a cardiotonic, was applied in the clinics for internal use at the dose of 1 tablespoon of the infusion (7:200) 3–5 times per day (Sokolov et al., 2000; Shikov et al., 2014).

Ten species are distributed in China. One thousand years ago, plants belonging to the *Adonis* genus in China (Chinese name: Binglianghua or Fushoucao) were recorded in the ancient book “Gui Hai Yu Heng Zhi” written by Fan Chengda, a notable historical figure from the Song dynasty. The well-known classical book of Chinese materia medica, “Ben Cao Gang Mu,” also noted the effect (Keshan Research Group of Jilin Medical University, First Clinical College, Second Clinical College, Third Clinical College of Jilin Medical University, 1977), and raw materials have been used in folk medicine for the treatment of heart diseases and edema (Bae, 2000). During the 1950s, due to the shortage of cardiac agents, *Adonis* sp. distributed throughout China were widely studied and developed. These efforts resulted in the isolation and further study of the cardenolide-enriched extracts of *A. amurensis*. After comprehensive pharmacological tests, the extracts were prepared and developed in a new preparation that was used to clinically treat human heart failure (Coronary Disease Control Group of Liaoning TCM College’s Hospital, 1971). In 1975, the raw material of this plant was listed in the Pharmacopeia of the People’s Republic of China (Committee for the Pharmacopoeia of P. R. China, 1975). In Siberia, the aqueous extract of the aerial parts was used to treat malaria, kidney disease and other heart-related diseases (Utkin, 1931; Nosal and Nosal, 1960).

## Phytochemistry

**FIGURE 2:**
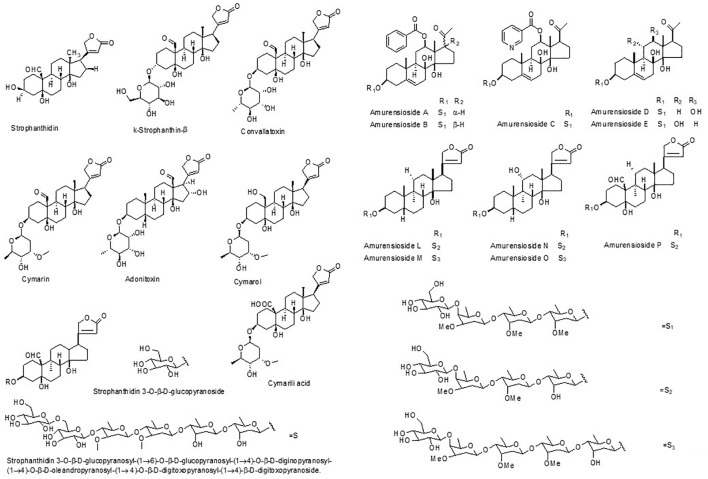
The chemical structure of some active compounds from the genus *Adonis.*

Since the first compound was isolated from *Adonis* plants in the early 19th century, more than 120 compounds have been isolated and identified to date. Fifty-four cardiac glycoside compounds were identified as active components. Additionally, flavones, carotenoids, coumarins and other compounds were also isolated and reported (Table 2). The chemical structures of active compounds isolated from the genus *Adonis* were listed in Figure 2.

**Table 2:** The isolated compounds from the genus *Adonis.*

No.	Compounds	Species	Reference
**Cardiac glycosides**			
(1)	Cymarin	*A. vernalis*	Katz and Reichstein, 1947; Ponomarenko et al., 1971a; Genkina et al., 1972; Komissarenko et al., 1973a,b,c, 1977; Lamzhav, 1975; Evdokimov, 1979; Ma et al., 1985; You et al., 2003; Yin et al., 2014
		*A. amurensis*	
		*A. wolgensis*	
		*A. chrysocyathus*	
		*A. tianschanicus*	
		*A. turkestanicus*	
		*A. leiosepala*	
		*A. mongolica*	
		*A. pseudoamurensis*	
(2)	Adonitoxin	*A. vernalis*	Katz and Reichstein, 1947; Lamzhav, 1975; Yatsyuk et al., 1976
		*A. chrysocyathus*	
		*A. mongolica*	
(3)	16-Hydroxy-strophanthidin	*A. vernalis*	Pitra and Čekan, 1961
(4)	Acetyladonitoxin	*A. vernalis*	Pitra and Čekan, 1961
(5)	Vernadigin	*A. vernalis*	Poláková and Čekan, 1965
(6)	3-Acetylstrophadogenin	*A. vernalis*	Poláková and Čekan, 1965
(7)	Substance N	*A. vernalis*	Büchner et al., 1965
(8)	Strophanthidine fucoside	*A. vernalis*	Wichtl et al., 1972
(9)	3-Epi-periplogenin	*A. vernalis*	Mathe and Mathe, 1979a,b; Junior and Wichtl, 1980; Kopp et al., 1992
		*A. aleppica*	
		*A. aestivalis*	
(10)	17β-(2′,5′-dihydro-5′-oxo-3′-furyl)-5β-14β-androstane-3α,5β,14β-triol	*A. vernalis*	Mathe and Mathe, 1979a,b
(11)	Adonitoxigenin 2-*O*-acetylrhamnosidoxyloside	*A. vernalis*	Peter and Max, 1980
(12)	Adonitoxigenin 3-*O*-acetylrhamnosidoxyloside	*A. vernalis*	Winkler and Wichtl, 1985
(13)	Adonitoxigenin rhamnosidoxyloside	*A. vernalis*	Winkler and Wichtl, 1985
(14)	Adonitoxigenin 3-*O*-[β-D-glucopyranosyl-(1→4)-α-L- rhamnopyranoside	*A. vernalis*	Winkler and Wichtl, 1986
(15)	Adonitoxigenin 3-*O*-[β-D-glucopyranosyl-(1→4)-α-L-(3′-*O*-acetyl)-rhamnopyranoside	*A. vernalis*	Winkler and Wichtl, 1986
(16)	Adonitoxigenin-3-[*O*-α-L(2′-*O*-acetyl) rhamnosido-β-D- glucoside	*A. vernalis*	Winkler and Wichtl, 1986
(17)	17β-(2′,5′-dihydro-5′-oxo-3′-furyl)-5β-14β-androstane-3α, 5β,14β-triol	*A. vernalis*	Junior and Wichtl, 1980
(18)	Periplorhamnoside	*A. aleppica*	Junior and Wichtl, 1980
		*A. amurensis*	Yin et al., 2014
(19)	Strophanthidin-diginoside	*A. aleppica*	Junior and Wichtl, 1980
(20)	Uzarigenin-3-*O*-sulfate	*A. aleppica*	Pauli and Junior, 1993
(21)	Alepposide A	*A. aleppica*	Pauli and Junior, 1993; Pauli, 1995
(22)	Alepposide B	*A. aleppica*	Pauli and Junior, 1993; Pauli, 1995
(23)	Alepposide C	*A. aleppica*	Pauli and Junior, 1993; Pauli, 1995
(24)	Alepposide D	*A. aleppica*	Pauli and Junior, 1993; Pauli, 1995
(25)	Sarmentocymarin	*A. aleppica*	Pauli and Junior, 1993; Pauli, 1995
(26)	Aleppotrioloside	*A. aleppica*	Matthiesen et al., 1992
(27)	Somalin	*A. amurensis*	Ma et al., 1985; Yin et al., 2014
		*A. pseudoamurensis*	
(28)	Cymarol	*A. amurensis*	You et al., 2003
(29)	Strophanthidin	*A. amurensis*	Ponomarenko et al., 1971a; Genkina et al., 1972; Komissarenko et al., 1973a,b,c, 1977; Zheng, 1975; Yatsyuk et al., 1983; Yin et al., 2014
		*A. aestivalis*	
		*A. wolgensis*	
		*A. chrysocyathus*	
		*A. sibiricus*	
		*A. tianschanicus*	
		*A. turkestanicus*	
(30)	Strophanthidol	*A. amurensis*	Ponomarenko et al., 1971a
**Cardiac glycosides**			
(31)	Corchoroside A	*A. amurensis*	Ponomarenko et al., 1971a; Lamzhav, 1975
		*A. mongolica*	
(32)	Convallatoxin	*A. amurensis*	Ponomarenko et al., 1971a; Komissarenko et al., 1973a,b,c; Zheng, 1975; Ma et al., 1985; Yin et al., 2014
		*A. wolgensis*	
		*A. sibiricus*	
		*A. pseudoamurensis*	
(33)	k-Strophanthin-β	*A. amurensis*	Ponomarenko et al., 1971a; Genkina et al., 1972; Komissarenko et al., 1973a,b,c, 1977; Lamzhav, 1975; Zheng, 1975; Evdokimov, 1979; Yatsyuk et al., 1983
		*A. aestivalis*	
		*A. wolgensis*	
		*A. chrysocyathus*	
		*A. sibiricus*	
		*A. tianschanicus*	
		*A. turkestanicus*	
		*A. leiosepala*	
		*A. mongolica*	
(34)	Digitoxigenin	*A. amurensis*	Sato et al., 1971; Yin et al., 2014
		*A. vernalis*	
(35)	Convalloside	*A. amurensis*	Yin et al., 2014
(36)	Amurensioside L	*A. amurensis*	Kubo et al., 2015
		*A. multiflora*	Baek et al., 2015
(37)	Amurensioside M	*A. amurensis*	Kubo et al., 2015
(38)	Amurensioside N	*A. amurensis*	Kubo et al., 2015
(39)	Amurensioside O	*A. amurensis*	Kubo et al., 2015
(40)	Amurensioside P	*A. amurensis*	Kubo et al., 2015
(41)	Cymarilic acid	*A. amurensis*	You et al., 2003
(42)	Helveticoside	*A. aestivalis*	Kopp et al., 1992
(43)	Strophanthidin-3-*O*-β-D-digitoxosido-α-L-cymarosido-β-D-glucoside	*A. aestivalis*	Kopp et al., 1992
(44)	Strophanthidin-3-*O*-β-D-digitoxosido-β-D-digitoxosido-β-D-diginosido-β-D-glucoside	*A. aestivalis*	Kopp et al., 1992
(45)	3β,5α,14β,17β-Tetrahydroxycard-20,22-enolide	*A.aestivalis*	Kubo et al., 2012
(46)	3β-[(*O*-β-D-glucopyranosyl) oxy]-5α,14β,17β-trihydroxycard-20(22)-enolide	*A. aestivalis*	Kubo et al., 2012
(47)	3β-[(*O*-β-D-Glucopyranosyl-(1→4)-*O*-β-D-glucopyranosyl) oxy]-5α,14β,17β-trihydroxycard-20(22)-enolide	*A. aestivalis*	Kubo et al., 2012
(48)	Strophanthidin 3-*O*-β-D-glucopyranosyl-(1→6)-*O*-β-D-glucopyranosyl-(1→4)-*O*-β-D-diginopyranosyl-(1→4)-*O*-β-D-oleandropyranosyl-(1→4)-*O*-β-D-digitoxopyranosyl-(1→4)-β-D-digitoxopyranoside	*A. aestivalis*	Kubo et al., 2012
(49)	Strophanthidin 3-*O*-β-D- glucopyranoside	*A. aestivalis*	Kubo et al., 2012
(50)	k-Strophanthoside	*A. chrysocyathus*	Yatsyuk et al., 1976
(51)	Gxtuagoxin	*A. sibiricus*	Zheng, 1975
(52)	Erysimoside	*A. mongolica*	Lamzhav, 1975
(53)	Olitoroside	*A. mongolica*	Lamzhav, 1975
(54)	Glucoolitoroside	*A. mongolica*	Lamzhav, 1975
**Other glycosides**			
(55)	Adonilide	*A. amurensis*	Shimizu et al., 1967, 1969a,b; Sato et al., 1971
		*A. vernalis*	
(56)	Fukujusone ester A	*A. amurensis*	Shimizu et al., 1967, 1969a,b
(5)7	Fukujusone ester B	*A. amurensis*	Shimizu et al., 1967, 1969a,b
(58)	Fukujusonorone	*A. amurensis*	Shimizu et al., 1967, 1969a,b; Sato et al., 1971
		*A. vernalis*	
(59)	Fukujusone	*A. vernalis*	Sato et al., 1971
		*A. amurensis*	
(60)	12-*O*-Nicotinoylisolineolon (Lineolon)	*A. vernalis*	Sato et al., 1971
		*A. amurensis*	
(61)	12-*O*-Benzoylisolineolon	*A. vernalis*	Sato et al., 1971
		*A. amurensis*	
(62)	Nicotinoylisoramanone	*A. vernalis*	Sato et al., 1971
		*A. amurensis*	
(63)	Isoramanone (digipurprogenin-II)	*A. vernalis*	Sato et al., 1971
		*A. amurensis*	
(64)	Isolineolon	*A. amurensis*	Shimizu et al., 1978
(65)	Amurensioside A	*A. amurensis*	Kuroda et al., 2010
(66)	Amurensioside B	*A. amurensis*	Kuroda et al., 2010
(67)	Amurensioside C	*A. amurensis*	Kuroda et al., 2010
(68)	Amurensioside D	*A. amurensis*	Kuroda et al., 2010
(69)	Amurensioside E	*A. amurensis*	Kuroda et al., 2010
(70)	Amurensioside F	*A. amurensis*	Kuroda et al., 2010
(71)	Amurensioside I	*A. amurensis*	Kuroda et al., 2010
(72)	Amurensioside G	*A. amurensis*	Kuroda et al., 2010
(73)	Amurensioside H	*A. amurensis*	Kuroda et al., 2010
(74)	Amurensioside J	*A. amurensis*	Kuroda et al., 2010
(75)	Amurensioside K	*A. amurensis*	Kuroda et al., 2010
**Flavones**			
(76)	Adonivernith (luteolin-8-hexityl monoxyloside)	*A. vernalis*	Drozd et al., 1971
		*A. leiosepala*	Evdokimov, 1979
		*A. tianschanicus*	
		*A. turkestanicus*	Komissarenko et al., 1977
(77)	Homoadonivernith	*A. vernalis*	Drozd et al., 1971
(78)	Orientin	*A. vernalis*	Wagner et al., 1975
		*A. coerulea*	Zhang et al., 1991
		*A. amurensis*	Yin et al., 2014
		*A. sibiricus*	Zheng, 1975
		*A. wolgensis*	Komissarenko et al., 1973c
		*A. tianschanicus*	
		*A. turkestanicus*	Komissarenko et al., 1977
(79)	Homoorientin	*A. vernalis*	Wagner et al., 1975
(80)	Isoorientin	*A. vernalis*	Wagner et al., 1975
		*A. coerulea*	Dai et al., 2010
(81)	Luteolin	*A. vernalis*	Budzianowski et al., 1991
		*A. coerulea*	Dai et al., 2010
		*A. mongolica*	Lamzhav, 1975
		*A. amurensis*	Yin et al., 2014
(82)	Vitexin	*A. vernalis*	Budzianowski et al., 1991
(83)	Apigenin	*A. coerulea*	Zhang et al., 1991
		*A. amurensis*	Yin et al., 2014
(84)	Luteolin 7-glucoside	*A. coerulea*	Dai et al., 2010
		*A. mongolica*	Lamzhav, 1975
(85)	Kaempferol	*A. mongolica*	Lamzhav, 1975
(86)	Orientin β-glucoside	*A. mongolica*	Lamzhav, 1975
(87)	Apigenin-7-*O*-β-D-glucuronide	*A. amurensis*	Yin et al., 2014
(88)	Isoquercitrin	*A. amurensis*	Yin et al., 2014
(89)	Calendula	*A. sibiricus*	Zheng, 1975
**Carotenoid**			
(90)	Astaxanthin	*A. annua*	Egger, 1965
		*A. aestivalis*	Kamata and Simpson, 1987
		*A. amurensis*	Zhang et al., 2015
(91)	Hydroxyechinenon	*A. annua*	Egger, 1965
(92)	Adonirubin	*A. annua*	Egger, 1965
(93)	Adonixanthin	*A. annua*	Egger, 1965
(94)	3,4-Dikcto-β-carotene	*A. annua*	Egger and Kleinig, 1967b
(95)	3,4,4′-Trikcto-β-carotene	*A. annua*	Egger and Kleinig, 1967b
(96)	Astaxanthin ester	*A. annua*	Egger and Kleinig, 1967a
(97)	3-Hydroxyechinenone ester	*A. annua*	Egger and Kleinig, 1967a
(98)	3,3′-Dihydroxyechinenone ester	*A. annua*	Egger and Kleinig, 1967a
(99)	3-Hydroxycanthaxanthin ester	*A. annua*	Egger and Kleinig, 1967a
(100)	Adonixanthin diester	*A. annua*	Renstrøm et al., 1981
(101)	3-Hydroxy-echinenone ester	*A. annua*	Renstrøm et al., 1981
(102)	*Cis*-astaxanthin diester	*A. annua*	Renstrøm et al., 1981
(103)	*Trans*-astaxanthin diester	*A. annua*	Renstrøm et al., 1981
(104)	Adonirubin ester	*A. annua*	Renstrøm et al., 1981
(105)	*Cis*-astaxanthin monoester	*A. annua*	Renstrøm et al., 1981
(106)	*Trans*-astaxanthin monoester	*A. annua*	Renstrøm et al., 1981
**Coumarins**			
(107)	Umbelliferone	*A. amurensis*	Ponomarenko et al., 1971b
		*A. wolgensis*	Komissarenko et al., 1973c
		*A. leiosepala*	Evdokimov, 1979
		*A. mongolica*	Lamzhav, 1983
(108)	Scopoletin	*A. amurensis*	Ponomarenko et al., 1971b
		*A. wolgensis*	Komissarenko et al., 1973c
		*A. leiosepala*	Evdokimov, 1979
		*A. mongolica*	Lamzhav, 1983
**Others**			
(109)	Linolenic acid	*A. wolgensis*	Mohadjerani et al., 2014
(110)	Oleic acid	*A. wolgensis*	Mohadjerani et al., 2014
(111)	Stigmast-4-ene-3,6-dione	*A. coerulea*	Zhang et al., 1991
(112)	Stigmast-4-ene-3-one 6β-hydroxy	*A. coerulea*	Zhang et al., 1991
(113)	β-D-glucopyranoside	*A. coerulea*	Zhang et al., 1991
(114)	Palmitic acid	*A. coerulea*	Zhang et al., 1991
(115)	Adonitol	*A. coerulea*	Zhang et al., 1991
		*A. mongolica*	Evdokimov, 1979
		*A. leiosepala*	Evdokimov, 1979
(116)	β-Sitosterol	*A. coerulea*	Zhang et al., 1991
(117)	1-Hentriacontanol,	*A. coerulea*	Dai et al., 2010
(118)	*P*-formylcinnamic acid	*A. coerulea*	Dai et al., 2010
(119)	Sugoroside	*A. chrysocyathus*	Genkina et al., 1972
(120)	Adoligose A	*A. aleppica*	Pauli, 1995
(121)	Adoligose B	*A. aleppica*	Pauli, 1995
(122)	Adoligose C	*A. aleppica*	Pauli, 1995
(123)	Adoligose D	*A. aleppica*	Pauli, 1995
(124)	Adoligose E	*A. aleppica*	Pauli, 1995
(125)	Pinoresinol	*A. amurensis*	Yin et al., 2014
(126)	Pinoresinol-8-*O*-β-D-glucopyranoside	*A. amurensis*	Yin et al., 2014
(127)	9′-Decarboxy rosmarinic acid-4′-*O*-(1→4)-galactosyl rhamnoside	*A. amurensis*	Yin et al., 2014


### Cardiac Glycosides and Other Glycosides

#### Cardiac Glycosides

Cardiac glycosides are important active compounds of the genus *Adonis*. Since the extract of *A. vernalis* was introduced into medicine in 1879, the increasing numbers of compounds have been isolated and identified. In 1918, a method for the preparation of an active digitalis-like glucoside from *A. vernalis* was developed (Heyl et al., 1918). Cymarin (1), adonitoxin (2), 16-hydroxy-strophanthidin (3), acetyladonitoxin (4), vernadigin (5) and 3-acetylstrophadogenin (6) were subsequently isolated (Katz and Reichstein, 1947; Pitra and Čekan, 1961; Poláková and Čekan, 1965). In 1965, a new glycoside, substance N (7), was isolated from the leaves of *A. vernalis* (Büchner et al., 1965). Additional isolated compounds include strophanthidine fucoside (8), 3-*epi*-periplogenin (9), 17β-(2′,5′-dihydro-5′-oxo-3′-furyl)-5β-14β-androstane-3α,5β,14β-triol (10), adonit oxigenin 2-*O*-acetylrhamnosidoxyloside (11), adonitoxigenin 3-*O*-acetylrhamnosidoxyloside (12), adonitoxigenin rhamnosid oxyloside (13) and cymarin (Franz, 1971; Wichtl et al., 1972; Mathe and Mathe, 1979a,b; Junior and Wichtl, 1980; Winkler and Wichtl, 1985). Adonitoxigenin 3-*O*-[β-D-glucopyranosyl-(1→4)-α-L-rhamno pyranoside (14), adonitoxigenin 3-*O*-[β-D-glucopyranosyl-(1→4)-α-L-(3′-*O*-acetyl)-rhamnopyranoside (15), adonit oxigenin-3-[*O*-α-L(2′-*O*-acetyl) rhamnosido-β-D-glucoside (16) and 17β-(2′,5′-dihydro-5′-oxo-3′-furyl)-5β-14β-androstane-3α, 5β,14β-triol (17) were also identified (Junior and Wichtl, 1980; Winkler and Wichtl, 1986).

*Adonis aleppica* is endemic in Mesopotamia and southeastern Anatolia and is closely related to *A. vernalis*, which is used as a heart tonic. In 1985, 3-*epi*-periplogenine, periplorhamnoside (18) and strophanthidin-diginoside (19) were isolated (Junior and Wichtl, 1980). Subsequently, the first cardenolide-sulfate uzarigenin-3-*O*-sulfate (20) was identified, along with alepposides A (21), B (22), C (23), and D (24); sarmentocymarin (25); and a glycosidic conjugate named aleppotrioloside (26) that were also isolated from the whole plant (Matthiesen et al., 1992; Pauli and Junior, 1993; Pauli, 1995).

Investigation of the chemical constituents of *A. amurensis* roots has been on-going since the 1960s, with more than 20 pregnanes and cardenolides isolated and identified. In 1971, eight cardenolides were isolated by Ponomarenko et al. (1971a), including cymarin (1), somalin (27), cymarol (28), strophanthidin (29), strophanthidol (30), corchoroside A (31), convallatoxin (32) and k-strophanthin-β (33). Subsequently, digitoxigenin (34) and convalloside (35) were identified from this plant (Shimizu et al., 1978; Yin et al., 2014). Kubo et al. (2015) isolated five new cardenolide glycosides, amurensiosides L-P (36–40). In 2003, antiangiogenic activity-guided fractionation and isolation carried out on the methanol extract of *A. amurensis* led to the identification of three compounds, namely, cymarin, cymarol, and cymarilic acid (41) (You et al., 2003). Digitoxigenin (34) was isolated from both *A. vernalis* and *A. amurensis* (Sato et al., 1971).

*Adonis aestivalis* is an annual plant with a crimson flower, distributed throughout southern Europe and Asia. Yatsyuk et al. (1983) first investigated the epigeal phytochemicals of *A. aestivalis*, which included strophanthidin and k-strophanthidin-β. In 1992, four cardenolides were isolated for the first time from the aerial parts of the plants, including 3-*epi*-periplogenin, helveticoside (42), strophanthidin-3-*O*-β-D-digitoxosido-α-L-cymarosido-β-D-glucoside (43) and strophanthidin-3-*O*-β-D-digitoxosido-β-D-digitoxosido-β-D-dig inosido-β-D-glucoside (44); the first two compounds have been isolated from other species as well (Kopp et al., 1992). Kubo et al. (2012) has investigated the chemical compounds in the seeds of *A. aestivalis*, and a new cardenolide 3β,5α,14β,17β-tetrahydroxycard-20,22-enolide (45) was found along with its two new glycosides 3β-[(*O*-β-D-glucopyranosyl)oxy]-5α,14β,17β-trihydroxycard-20(22)-enolide (46), and 3β-[(*O*-β-D-glucopyranosyl-(1→4)-*O*-β-D-glucopyranosyl)oxy]-5α,14β,17β-trihydroxycard-20(22)-enolide (47). A new strophanthidin hexaglycoside, strophanthidin 3-*O*-β-D-glucopyranosyl-(1→6)-*O*-β-D-glucopyranosyl-(1→4)-*O*-β-D-diginopyranosyl-(1→4)-*O*-β-D-oleandropyranosyl-(1→4)-*O*-β-D-digitoxopyranosyl-(1→4)-β-D-digitoxopyranoside (48), as well as strophanthidin 3-*O*-β-D-glucopyranoside (49) were also isolated (Kubo et al., 2012).

*A. multiflora* is native to Korea, Japan, and Manchuria. In 2015, amurensioside L (36) was isolated from the whole plant (Baek et al., 2015). *A. leiosepala* yielded cymarin and k-strophanthin-β (Evdokimov, 1979). These two compounds, along with strophanthidin and convallatoxin, were isolated from *A. wolgensis* (Komissarenko et al., 1973a,b,c). Strophanthidin, cymarin, k-strophanthin-β, k-strophanthoside (50) and adonitoxin were identified in extracts of *A. chrysocyathus* (Aitova et al., 1971; Genkina et al., 1972; Yatsyuk et al., 1976). Then, the related plant *A. sibiricus* afforded strophanthidin, k-strophanthidin-β, convallatoxin and gxtuagoxin (51) (Zheng, 1975).

Lamzhav (1975) isolated cymarin, adonitoxin, corchoroside A, k-strophanthin-β, k-strophanthoside, erysimoside (52), olitoroside (53) and glucoolitoroside (54) from *A. mongolica* (Thieme and Lamzhav, 1976). Komissarenko et al. (1977) isolated the cardenolides strophanthidin, cymarin and k-strophanthin-β from *A. tianschanicus* and *A. turkestanicus.* Finally, somalin, cymarin, and Convallatoxin were identified in *A. pseudoamurensis* (Ma et al., 1985).

#### Other Glycosides

Shimizu et al. (1967, 1969a,b) identified an aglycone-adonilide (55); three novel compounds, namely, fukujusone, ester A (56) and ester B (57); and the 18-norpregnane derivative fukujusonorone (58) in *A. amurensis*. Adonilide (55), fukujusone (59), 12-*O*-nicotinoylisolineolon (lineolon, 60), 12-*O*-benzoylisolineolonb (61) and fukujusonorone (58), together with nicotinoylisoramanone (62), digitoxigenin, and isoramanone (digipurprogenin-II, 63) were isolated from *A. vernalis* and *A. amurensis* (Sato et al., 1971). Isolineolon (64) was also isolated from this plant (Shimizu et al., 1978).

In 2010, five new pregnane tetraglycosides known as amurensiosides A–E (65–69); two new pregnane hexaglycosides, amurensiosides F (70) and I (71); two new 18-norpregnane hexaglycosides, amurensiosides G (72) and H (73); and two new pregnane octaglycosides, amurensiosides J (74) and K (75), were isolated from the MeOH extracts of the roots of *A. amurensis* (Kuroda et al., 2010). A new pregnane hexaglycoside was isolated from the whole plant (Baek et al., 2015).

##### Flavones

Along with the isolated cardiac compounds, many flavones were also identified. Adonivernith (luteolin-8-hexityl monoxyloside) (76), homoadonivernith (77), orientin (78), homoorientin (79), isoorientin (80), luteolin (81) and vitexin (82) were isolated from *A. vernalis* (Chernobai et al., 1968; Drozd et al., 1971; Wagner et al., 1975; Budzianowski et al., 1991), and adonivernith also was found in *A. leiosepala* (Evdokimov, 1979).

Orientin, apigenin (83), luteolin, isoorientin and luteolin 7-glucoside (84) were isolated from *A. coerulea* Maxim. (Zhang et al., 1991; Dai et al., 2010). Lamzhav (1975, 1983) isolated luteolin, kaempferol (85), luteolin 7-glucoside, and an orientin β-glucoside (86) from *A. mongolica*, and luteolin, apigenin, apigenin-7-*O*-β-D-glucuronide (87), orientin and isoquercitrin (88) were found in *A. amurensis* (Yin et al., 2014). Orientin was identified from *A. sibiricus* (Zheng, 1975). Komissarenko et al. (1973c) has identified the flavonoid orientin from *A. wolgensis*, while the orientin and adonivernitol were isolated from the herbs *A. tianschanicus* and *A. turkestanicus* (Komissarenko et al., 1973b, 1977).

##### Carotenoids

In 1965, astaxanthin (90), along with three minor red compounds known as hydroxyechinenon (91), adonirubin (4,4′-diketo-3-hydroxy-β-carotene) (92) and adonixanthin (3,3′-hydroxy-4-keto-β-carotene) (93) were identified from the red flowers of *A. annua* (Egger, 1965). Astaxanthin also was found in *A. amurensis* (Zhang et al., 2015). 3,4-Diketo- and 3,4,4′-triketo-β-carotene (94, 95) were also isolated (Egger and Kleinig, 1967b). The fatty acid components of the ketocarotenoid esters, including esters of astaxanthin (96), 3-hydroxyechinenone (97), 3,3′-dihydroxyechinenone (98) and 3-hydroxycanthaxanthin (99) were also investigated (Egger and Kleinig, 1967a). In 1981, the carotenoid composition of the red flower petals of *A. annua* was elucidated and included adonixanthin diester (100), 3-hydroxy-echinenone ester (101), *cis*-astaxanthin diester (102), *trans*-astaxanthin diester (103), adonirubin ester (104), *cis*-astaxanthin monoester (105) and *trans*-astaxanthin monoester (106) (Renstrøm et al., 1981). In1987, from *A. aestivalis* astaxanthin diester also was isolated (Kamata and Simpson, 1987).

##### Coumarins

The two coumarins umbelliferone (107) and scopoletin (108) were isolated from the roots of *A. amurensis*, *A. wolgensis, A. leiosepala*, and *A. mongolica* (Ponomarenko et al., 1971b; Komissarenko et al., 1973c; Evdokimov, 1979; Lamzhav, 1983).

##### Others

Mohadjerani et al. (2014) studied the fatty acids of *A. wolgensis*, and the results showed that linolenic acid (45.83%, 109) and oleic acid (47.54%, 110) were the most abundant fatty acids found in the leaves and stems, respectively. Zhang et al. (1991) found that stigmast-4-ene-3,6-dione (111), stigmast-4-ene-3-one 6β-hydroxy (112), β-D-glucopyranoside (113), palmitic acid (114), adonitol (115), and β-sitosterol (116) existed in *A. coerulea*. 1-Hentriacontanol (117) and *p*-formylcinnamic acid (118) were also found in this plant (Dai et al., 2010).

A new tetraoside, sugoroside (119) was identified in the extracts of *A. chrysocyathus* (Genkina et al., 1972), and the pentahydric alcohol adonitol was found in *A. mongolica* and *A. leiosepala* (Evdokimov, 1979). Five novel tri-, tetra-, and penta-saccharides named adoligoses A-E (120–124), consisting of rare dideoxy sugars and their 3-OMe ethers, have been isolated from *A. aleppica* (Pauli, 1995).

Three lignans, namely, pinoresinol (125), pinoresinol-8-*O*-β-D-glucopyranoside (126) and 9′-decarboxy rosmarinic acid-4′-*O*-(1→4)-galactosyl rhamnoside (127), were isolated from *A. amurensis* (Yin et al., 2014).

## Analysis of Active Constituents and Quality Control

Due to the marked cardiac-enhancing effects, *Adonis* spp. have long been used in European and Chinese folk medicine, and some species, such as *A. amurensis*, have been historically applied in the clinic to treat heart diseases. To examine the active compound content in different parts of the plants and in different species, high-performance liquid chromatography (HPLC) and other chromatographic methods were utilized. Wang et al. (1991) reported that the highest content of total cardenolide glycosides was found in the roots of *A. amurensis* during the germination period with the lowest content levels isolated during the mature fruit phase. Chromatography of cardiac glycosides in *A. amurensis* used CH_3_OH: H_2_O (65:35) as the mobile phase with an ODS column (150 mm × 6.0 mm) at a flow rate of 0.80 mL/min monitored at 218 nm. The contents of convallatoxin, strophanthidin, cymarin, and aglycones A and B found in cardenolide-enriched extract were 6.58, 2.09, 2.54, 4.49, and 2.11%, respectively, in a chloroform-ethanol (1:1) fraction of an ethanolic extract (Gu et al., 1990). Liu and Cui (2007) studied the content of convallatoxin of *A. amurensis* obtained from various habitats throughout China. The results quantified the contents of the aerial parts and roots harvested from Liaoning province (0.0022 and 0.1400%), Jilin province (0.0019 and 0.1300%) and Heilongjiang province (0.0014 and 0.0790%). The amounts of somalin, k-strophanthoside and k-strophanthin-β in *A. pseudoamurensis* were determined to be 0.024, 0.13, and 0.071%, respectively (Gu et al., 1989).

## Pharmacology

### Cardiovascular Effect

In 1918, the cardiovascular effect and the toxicity was firstly assayed using the 1-h frog method. Results showed that at the concentration of 0.0045 mL/g frog of ten percent of 95% ethanol extract of *A. vernalis* could result in a permanent systole (M.S.D.) of the frog’s ventricle at the end of 1 h (Heyl et al., 1918). In the early 1930s, Munch and Krantz (1934) reported that *A. vernalis* and its preparations exhibited the same level of potency in the heart as digitalis and the corresponding digitalis preparations using the 1-h frog method. Studies by Benson and Edwards (1941) showed that the pigeon emetic method is suitable for the assay of *A. vernalis*, and the percent potency of tincture of *Adonis* assays was 100% by the frog method, 91.6% by the cat method, and 85.37% by the pigeon emetic method. Subsequently, Lehmann (1984) studied the cardiac inotropic and constrictor of SCOA (contained extracts from *Scilla, Convallaria, Oleander*, and *Adonis*) in cats *in vivo*. At the dose of 21.5–100 GPU/kg (GPU, guinea-pig unites), SCOA after intravenous injection had a positive inotropic and constrictor effect on veins and arteries. According to the studies of Turova and Sapozhnikova (1989), the raw material of *Adonis* is as effective as *Digitalis* in the heart failure accompanied by cardiac conduction disturbance; but the effects are not cumulative and could not result in the phenomenon of a cardiac arrest caused by *Digitalis.* Meanwhile, substance N (7) from the leaves of *A. vernalis* exhibited a highly potent digitalis-like mode-of-action, with a geometrical mean LD of 0.1141 ± 0.0040 mg/kg in cats (Büchner et al., 1965). Moreover, the potent antihyperlipidemic activity of the alcoholic extract of *A. vernalis* also was found. At the concentration of 5 mg/kg, it could significant decrease the serum cholesterol and triglycerides compared with control, triton-induced hyperlipidemic control and positive control (simvastatin, 20 mg/kg) (*p* < 0.05). And it also slightly increased HDL, clear decrease in LDL and total protein (Lateef et al., 2012).

Kuo et al. (1962) first studied the cardiac activity of *A. amurensis*. The results showed that it has a similar effect to *A. vernalis*, and it could enhance contractions of an isolated frog heart and increase the contractions and diastole of an isolated rabbit heart. After assaying for 20–30 min, the contractions and diastole became weak, and the heartbeat stopped at the systolic stage. Moreover, it enhanced the contractions of a dog heart and increased the blood pressure while decreasing the venous pressure of a heart in failure. Further electrocardiogram tests showed that it extended the P-Q interval and shortened the R-T interval, indicating that *A. amurensis* could influence metabolism of heart muscle, enhance heart muscle contractions, delay atrioventricular conduction and improve the overall function of the heart.

Additionally, the effect of the cardenolide-enriched extract on treatment of premature ventricular contraction was also reported (Dong, 1981). To investigate the mechanism of action for treating arrhythmia, the electrophysiology of rat cardiac muscle cells was studied. The results showed that after injecting 0.5 mg/kg cardenolide-enriched extract (0.5 mg/kg) in anesthetized rats (10% urethane, 0.5 g/kg), the repolarization action potential time limit (APD) lengthened, particularly at 90% APD, and the conduction velocity of action potential was slowed (Gu et al., 1981). When it was intravenously injected (0.1 mg/kg) in anesthetized dogs, the dp/dt max value increased significantly from 5 min to 30 min (*p* < 0.01), and this value was maintained after 1 h. In contrast to the value of dp/dt max, the heart rate of dogs significantly decreased (*p* < 0.001) immediately after injecting the extract, and while the time lengthened, the effect gradually weakened. Further studies showed the above trends did not change with administration of a β-receptor blocker, and this result indicated that β-receptor stimulation and release of endogenous catecholamine are not factors in the positive inotropic action of this extract. Additionally, the effect of myocardial potassium loss promoted by the total glycosides was presented in this research (Shi et al., 1979). The extract also enhanced the antiarrhythmic activity of disopyramide (Shen et al., 1983). To thoroughly exploit the resources of *A. amurensis*, the cardiotonic activities of the ethanol extract of leaves, stems, and roots were investigated. Results showed that all extracts exerted cardiotonic effects on the movement of a rabbit atrial muscle (Qin, 2000).

Deng et al. (1963) first proved that the total glycosides of *A. brevistyla*, found in the Yunan province of China, had cardiotonic effects. The results showed that injecting the total glycoside preparation could stop the muscle contraction of *Rana pleuraden* in the contraction phase when anesthetized with urethane and could enhance heart muscle contractions of rabbits after injections of 10% pentobarbital sodium. The LD_50_ value in pigeons was 7.08 ± 0.15 mg/kg.

The cardiotonic effects of cardenolide-enriched extract of *A. pseudoamurensis* on the heart failure of rabbits were studied and found to significantly improve the heart function in heart failure, while enhancing the dp/dt max, -dp/dt max, Co, and Lvsp of the heart with increased rates of 210 ± 33%, 70 ± 17%, 191 ± 51%, and 31 ± 30%, respectively (Chi et al., 1985). Oral administration of methyluracil lowered the sensitivity to strophanthin both in rabbits with myocardial infarction and in intact mice; intravenous administration of methyluracil increased the coronary circulation rate (Lazareva, 1975).

Maham and Sarrafzadeh-Rezaei (2014) reported the cardiovascular effects of *A. aestivalis* in anesthetized sheep. The results showed that after intravenously administering three successive equal doses (75 mg/kg) of the hydroalcoholic extract to anesthetized sheep, the extract induced significant bradycardia, hypotension, and various ECG abnormalities. Ventricular arrhythmias, bradyarrhythmias, atrioventricular blockage, premature ventricular beats, ventricular tachycardia, and ventricular fibrillation were observed. The acute intraperitoneal toxicity (LD_50_) of the extract in mice was 2150 mg/kg. The bradycardia and ECG alterations induced by the extract justified the traditional use of this plant in treating cardiovascular insufficiency (Table 3).

**Table 3:** Effects of the genus *Adonis* extracts and active compounds.

Effects	Species	Extracts or compounds	Dose	Results	Reference
Cardiovascular effect	*Adonis vernalis*	95% Ethanol extract	0.0045 mL/g frog	A permanent systole of the frog’s ventricle at the end of 1 h	Heyl et al., 1918
		No mentioned	–	Have the same level of potency in the heart as digitalis	Munch and Krantz, 1934
		No mentioned	–	The percent potency was 100, 91.6, and 85.37% by the frog, cat and pigeon method	Benson and Edwards, 1941
		No mentioned	21.5–100 GPU/kg (GPU, guinea-pig unites)	SCOA (*Scilla*, *Convallaria*, *Oleander*, and *Adonis*) in cats has positive inotropic and constrictor effect on veins and arteries	Lehmann, 1984
		No mentioned	–	It is as effective as Digitalis in the heart failure	Turova and Sapozhnikova, 1989
		Substance N	–	Highly potent digitalis-like mode-of-action,	Büchner et al., 1965
		Alcoholic extract	5 mg/kg	It significant decrease the serum cholesterol and triglycerides	Lateef et al., 2012
	*Adonis amurensis*	Cardenolide-enriched extract	–	It influences metabolism of heart muscle, enhance muscle contractions, delay atrioventricular conduction and improve the overall function of the heart	Kuo et al., 1962
		Cardenolide-enriched extract	0.5 mg/kg	It lengthened the repolarization action potential time limit and slowed the conduction velocity of action potential of rats	Gu et al., 1981
		Cardenolide-enriched extract	0.1 mg/kg.	It (i.v.) increased the dp/dt max value from 5 min to 30 min. The heart rate of dogs significantly decreased. It also promoted myocardial potassium loss.	Shi et al., 1979
		Cardenolide-enriched extract	–	It enhanced the antiarrhythmic activity of disopyramide.	Shen et al., 1983
		Ethanol extract	–	Extracts exerted cardiotonic effects on the movement of a rabbit atrial muscle.	Qin, 2000
	*Adonis brevistyla*	The total glycosides	–	It stopped the muscle contraction in the contraction phase and enhanced heart muscle contractions of rabbits.	Deng et al., 1963
	*Adonis pseudoamurensis*	Cardenolide-enriched extract	–	It improved the heart function in heart failure, while enhancing the dp/dt max, -dp/dt max, Co, and Lvsp with increased rates of 210, 70, 191, and 31%, respectively.	Chi et al., 1985
	*Adonis aestivalis*	Hydroalcoholic extract	75 mg/kg	It induced significant bradycardia, hypotension, and various ECG abnormalities. Ventricular arrhythmias, bradyarrhythmias, atrioventricular blockage, premature ventricular beats, and some abnormalities were observed.	Maham and Sarrafzadeh-Rezaei, 2014
Antiangiogenic effect	*Adonis amurensis*	Methanol extract	50 μg/mL	It exhibited strong inhibitory activity on human umbilical vein endothelial cells (HUVEC) tube formation.	Bae et al., 2000
	–	Cymarilic acid	1 μg/mL	It exhibited stronger inhibition of human umbilical venous endothelial (HUVE) cell-induced tube formation, with inhibition rates of 80–60% than cymarin and cymarol.	You et al., 2003
Cytotoxicity	–	Amurensioside A	–	It has cytotoxic to HSC-2 cells with IC_50_ value of 66 μg/mL.	Kuroda et al., 2010
	–	Amurensioside B	–	It has cytotoxic to HSC-2 cells with IC_50_ value of 26 μg/mL.	Kuroda et al., 2010
	–	Amurensioside D	–	It has cytotoxic to HSC-2 cells with IC_50_ values of 47 μg/mL.	Kuroda et al., 2010
	–	Amurensioside E	–	It has cytotoxic to HSC-2 cells with IC_50_ value of 58 μg/mL.	Kuroda et al., 2010
	–	3β-[(*O*-β-D-Glucopyranosyl) oxy]-5α,14β,17β-trihydroxycard-20(22)-enolide	–	The selective cytotoxicity toward malignant tumor cell lines including HSC-2, HSC-3, HSC-4, and HL-60 cells was 0.084–2.8 μM.	Kubo et al., 2012
	–	Strophanthidin 3-*O*-β-D-glucopyranosyl-(1→6)-*O*-β-D-glucopyranosyl-(1→4)-*O*-β-D-diginopyranosyl-(1→4)-*O*-β-D-oleandropyranosyl-(1→4)-*O*-β-D-digitoxopyranosyl-(1→4)-β-D- digitoxopyranoside	–	The selective cytotoxicity toward malignant tumor cell lines including HSC-2, HSC-3, HSC-4, and HL-60 cells was 0.086–0.55 μM.	Kubo et al., 2012
	–	Strophanthidin 3-*O*-β-D-glucopyranoside	–	The selective cytotoxicity toward above four malignant tumor cell lines was 0.012–0.062 μM.	Kubo et al., 2012
	–	Cymarin.	–	It has cytotoxicity against A549 cells (IC_50_ 0.031 μg/mL).	You et al., 2003
	–	Cymarol	–	It has potent cytotoxicity against A549 cells (IC_50_ 0.021 μg/mL).	You et al., 2003
Effect on the central nervous system	*Adonis amurensis*	The cardenolide-enriched extract	0.3 and 0.5 mg/kg	After injecting the extract (i.v.) in rabbits, the electroencephalogram has a high amplitude slow wave, and the response of rabbits to sound became weak. The spontaneous electro discharge in the neck was decreased, while the 5-HT content in the brain increased at 0.5 mg/kg.	Gu et al., 1980
Free radical scavenging capacity	*Adonis wolgensis*	Total phenolic content of the hydro-methanolic extract	–	It was 9.20 gallic acid equivalents/g dry matter. And an IC_50_ value of the free radical scavenging capacity was 27.45 μg/mL.	Mohadjerani et al., 2014
Antibacterial effect	*Adonis wolgensis*	The hydro-methanolic extract	–	It was effective against Gram-negative *Salmonella enteritidis* (48 μg/mL) and *Escherichia coli* (50 μg/mL) and against Gram-positive *Staphylococcus aureus* (50 μg/mL)	Mohadjerani et al., 2014
Anti-inflammatory effect	*Adonis vernalis*	Methanol extract	500 μg/mL	35% inhibition rate against tumor necrosis factor-α production in whole blood cell culture.	Das et al., 2007
Antiviral activity	*Adonis vernalis*	10% Aqueous extract	0.02 mL	Cytotoxic effect with inhibition zone 15–30 mm, virustatic effect with inhibition zone over 30 mm for all viruses	May and Willuhn, 1978
Diuretic effect	*Adonis amurensis*	The cardenolide-enriched extract	0.2 mg/kg	After injecting the extract into dogs, the average amount of urine measured increased to 178.03 mL. Na^+^, K^+^, and Cl^+^ outputs increased by 2.9-, 1.4-, and 1.9-fold compared to the control group, respectively.	Wang et al., 1981
Acaricidal activity	*Adonis coerulea*	Methanol extract	250 mg/mL	It presented acaricidal activity against *P. cuniculi* with LT_50_ of 3.137 h *in vitro*, and cured rabbit acariasis after three treatments. The mechanism of death in involved the destroyed motor function.	Shang et al., 2013, 2017.


### Antiangiogenic Activity

*Adonis amurensis* has been used in folk medicine for the treatment of several diseases such as cardiac insufficiency and edema (Bae, 2000), and the methanol extract was found to exhibit strong inhibitory activity on human umbilical vein endothelial cells (HUVEC) tube formation (Bae et al., 2000). The antiangiogenic activities of three compounds, namely, cymarin, cymarol, and cymarilic acid were studied. Among three compounds, cymarilic acid exhibited strong inhibition of human umbilical venous endothelial (HUVE) cell-induced tube formation, with inhibition rates of 80–60% at a concentration of 1 μg/mL. Cymarin and cymarol exhibited the same inhibitory activity against HUVE cells as the former compound (You et al., 2003) (Table 3).

### Cytotoxicity

In 2010, the cytotoxicity of four active compounds was found. Amurensioside A, amurensioside B, amurensioside D, and amurensioside E were moderately cytotoxic to HSC-2 cells with IC_50_ values of 66, 26, 47, and 58 μg/mL, respectively; the activity of the positive control melphalan was 13 μg/mL (Kuroda et al., 2010). 3β-[(*O*-β-D-glucopyranosyl)oxy]-5α,14β,17β-trihydroxycard-20(22)-enolide (46), strophanthidin 3-*O*-β-D-glucopyranosyl-(1→6)-*O*-β-D-glucopyranosyl-(1→4)-*O*-β-D-diginopyranosyl-(1→4)-*O*-β-D-oleandropyranosyl-(1→4)-*O*-β-D-digitoxopyranosyl-(1→4)-β-D-digitoxopyranoside (48), as well as strophanthidin 3-*O*-β-D-glucopyranoside (49) displayed selective cytotoxicity toward malignant tumor cell lines including HSC-2, HSC-3, HSC-4, and HL-60 cells with a CC_50_ range of 0.012–2.8 μM. Studies also indicated that they may trigger caspase-3-independent apoptotic cell death in HL-60 and HSC-2 cells. The CC_50_ values of the positive control melphalan were 8.7, 25, 32, and 1.4 μM in HSC-2, HSC-3, HSC-4, and HL-60 cells, respectively (Kubo et al., 2012). Five new cardenolide glycosides, amurensiosides L-P showed cytotoxic activities against HL-60 promyelocytic and HSC-2 cells (Kubo et al., 2015). Cymarin and cymarol showed potent cytotoxicity against A549 cells (0.031 and 0.021 μg/mL) while being inactive toward L1210 cells (5 μg/mL) (You et al., 2003). Cymarilic acid showed no significant cytotoxicity against the human solid tumor cell line A549 (ED_50_ > 5 μg/mL), and was inactive toward murine leukemic cells L1210 (ED_50_ > 5 μg/mL) (Table 3).

### Effect on the Central Nervous System

In 1980, Gu et al. (1980) studied the effect of the cardenolide-enriched extract of *A. amurensis* on the central nervous system of rabbits. After injecting the extract (0.3 and 0.5 mg/kg, i.v.) in rabbits, the electroencephalogram (EEG) presented a high amplitude slow wave, and the response of rabbits to sound became weak. The sedative effect of the total glycosides may be related to its inhibitory effect on the cerebral cortex and the reticular structure. Additionally, the spontaneous electro discharge in the neck was decreased, while the 5-HT content in the brain increased significantly at a concentration of 0.5 mg/kg. This result also showed that the glycosides induced peripheral muscle relaxation. Moreover, injecting the extract (5–15 μg) in the brain would stimulate the rabbits. Stimulation decreased when scopolamine (2 mg) was administered to rabbits (Table 3).

### Free Radical Scavenging Capacity

In 2014, the free radical scavenging capacity of *A. wolgensis* in DPPH radical scavenging assay was studied. Total phenolic content (TPC) of the hydromethanolic extract was 9.20 gallic acid equivalents/g dry matter. Studies showed that the free radical scavenging capacity of the hydro-methanolic extract had an IC_50_ value of 27.45 μg/mL, while the positive control ascorbic acid was 22.23 μg/mL. Additionally, the reducing potential of this extract (measured at 0.05–0.6 mg/mL) showed a general increase in activity with increasing concentration (Mohadjerani et al., 2014) (Table 3).

### Antibacterial, Anti-inflammatory, and Antiviral Activities

The hydro-methanolic extract of *A. wolgensis* was particularly effective against Gram-negative *Salmonella enteritidis* (48 ± 1.56 μg/mL) and *Escherichia coli* (50 ± 1.94 μg/mL) and against Gram-positive *Staphylococcus aureus* (50 ± 1.83 μg/mL), but no activity was observed against Gram-positive *Bacillus subtilis* (Mohadjerani et al., 2014). Das et al. (2007) reported a significant inhibitory effect by the 50% methanol extract of *A. vernalis* on tumor necrosis factor-α (TNF-α) production in whole blood cell culture. The 10% aqueous extract of *A. vernalis* aerial part also presented the antiviral activity with inhibition zone over 30 mm for Herpes virus Hominis HVP 75 (type2), influenza virus A2 (Manheim 57), Vaccini virus and poliovirus type1 (May and Willuhn, 1978) (Table 3).

### Diuretic Effect

Wang et al. (1981) found that the cardenolide-enriched extract of *A. amurensis* had a diuretic effect on dogs. After injecting the drug (0.2 mg/kg) into dogs, the average amount of urine measured increased to 178.03 mL versus 71.58 mL measured in the control group. Na^+^, K^+^, and Cl^+^ outputs increased by 2.9-, 1.4-, and 1.9-fold compared to the control group, respectively. These results indicated that the total glycoside preparation has a significant diuretic effect by inhibiting the renal tubular reabsorption of Na^+^, K^+^, and Cl^+^ (Table 3).

### Acaricidal Activity

*Adonis coerulea* is a perennial plant with a height of 2–12 cm, distributed throughout northeastern areas of Tibet and in Sichuan, Qinghai and Gansu Provinces in China at altitudes of 2300–5000 m (Chinese Materia Editorial Committee, and State Chinese Medicine Administration Bureau, 2002). In the field investigation of Sichuan and Gansu Provinces in China, *A. coerulea*, as a traditional Tibetan medicine to treat animal acariasis, was found (Shang et al., 2012). Further studies showed that the extract presented marked acaricidal activity against *Psoroptes cuniculi* with a median lethal time (LT_50_) of 3.137 h at a concentration of 250 mg/mL *in vitro*, and it cured rabbit acariasis after three treatments (Shang et al., 2013). The mechanism of death in *P. cuniculi* involved the inhibition of the dynamic equilibrium between the production and clearing of superoxide anions, which destroyed motor function (Shang et al., 2017) (Table 3).

## Toxicity

Animals consuming plants containing cardiac glycosides typically develop fatal digestive and cardiac disturbances (Galey et al., 1996), and many acute animal poisonings have been attributed to the *Adonis* spp. cardiac glycosides since 1912. These species include but are not limited to, *A. aestivalis*, *A. annua, A. amurensis, A. autumnalis*, and *A. microcarpa* (Maiden, 1912). The first experimental feeding trial was performed in 1929, and the results demonstrated that *A. annua* was lethal to sheep when fed 1.0 lb of fresh plant, the seed-bearing mature stage of the plant and extracts of the partially dried plant. However, feeding cattle 2 to 6 lb daily for 36 days failed to elicit clinical signs and death (Hurst, 1942). In 1932, toxicosis in horses was reported based on natural exposure to *Adonis* sp. (Degen, 1932; Kummer, 1952).

Woods et al. (2004) first reported *Adonis* toxicosis in North America. After eating grass hay containing *A. aestivalis*, three horses died. The signs of colic first appeared 24–48 h after initial exposure to the hay, and gastrointestinal stasis and myocardial degeneration of the horses were noted in subsequent clinical examinations. In 2007, the toxicity of *A. aestivalis* in calves was studied. Four Holstein and preruminating Jersey calves were administered 1% bodyweight of *A. aestivalis* (containing 11–98 mg/g of strophanthidin) via a stomach tube and monitored for clinical signs for 2 weeks and 1 week, respectively. The Holstein calves were then fed 0.2–1% bodyweight daily for 4–5 weeks. They had transient, mild cardiac abnormalities during the feeding trial, and mild transient gastrointestinal and cardiac signs were also noted in the preruminating calves. The above results showed that cattle are less susceptible than horses to cardiotoxic effects and sudden death after ingestion of relatively small quantities of *A. aestivalis* (Woods et al., 2007). Finally, the toxicity of *A. aestivalis* in sheep (ewes) was investigated in 2010. Results showed that after administrating 1% bodyweight to ewes for 24 and 48 h, the ewes all exhibited transient sinus arrhythmias, and two of the three ewes exhibited transient reduced fractional shortening. Moreover, after administering 0.2% bodyweight daily for 2 weeks, two ewes had reduced fractional shortening after the low-dose treatment regimen. No gross or microscopic lesions were seen when the ewes were examined postmortem at the end of the study (Woods et al., 2011).

In 1962, the toxicity of *A. amurensis* was first studied. After perfusing the cardenolide-enriched extract intravenously, the minimum lethal doses against cats and pigeons were 46.2 and 78.6 mg/kg, respectively (Kuo et al., 1962). In 1973, the toxicity to cats of the total glycoside preparation of *A. amurensis* was studied by observing the electrocardiogram, with results indicating that the minimum lethal dose in cats was 0.75 mg/kg (i.v.), while the minimum lethal doses of cedilanid and k-strophanthin were 0.77 and 0.49 mg/kg, respectively. The accumulative rates in body at 24 and 48 h were 74.2 and 23.8%, respectively, and at 74 h, the accumulative rate was less than 5%. The above results indicated the accumulative toxicity of the extract was lower than that of digitoxin and convallatoxin, higher than that of k-strophanthin (Shuguang Medical Team of Anshan City et al., 1973). The minimum lethal dose in pigeons was 1.469 ± 0.201 mg/kg (i.v.) (Shi et al., 1979). Acute toxicosis in mice and cats was also observed after intravenous administration of *Adonis*-like glycosides and the strophanthidin aglycone in the laboratory (Chen et al., 1951; Greeff and Kasperat, 1961).

Davies and Whyte (1989) found that feeding the seed of *A. microcarpa* (5.6 g/kg) induced total feed refusal within 3 days in growing and mature pigs, causing vomiting, rapid and shallow breathing, and even one pig died. These effects were probably caused by the cardiac glycosides and subsided within 2 weeks of removal of the seed. The toxicities of active compounds also were studied. The LD_50_ of cymarin after intravenous injection in rats and cats were 24.8 and 95.4 mg/g, respectively (Chen et al., 1942; Vogel and Kluge, 1961); and the LD_50_ for adonitoxin was 191.3 μg/kg (Chen and Anderson, 1947). Meanwhile, the average minimum dose producing a permanent systole (M.S.D.) values for above two compounds were 0.621 and 0.88 g/g frog, respectively (Chen and Anderson, 1947). After continuous intravenous infusion in dogs, the minimal lethal doses of adonidoside and adonivernoside at 30 min were found to be 0.7 and 1.75 mg/kg, respectively, and when they were used together, the LD_50_ was 1.14 mg/kg (Lenel-Pekelis, 1949). Kovaříková and Chen (1965) studied the activities of 16-hydroxy-strophanthidin, 16-formyloxy-strophanthidin, acetyladonitoxin, and tetracetyladonitoxin, and results showed that the LD_50_ in cats were 1.121, 0.1518, 0.3881, and 4.397 mg/kg, respectively.

In China, cases of *A. amurensis* poisoning in humans who misused or overdosed the plant have been noted. In most cases, the patient heart rate was seriously abnormal (Wang and Feng, 1982; Sun, 1988; Zhang, 1999).

## Conclusion and Remarks

Because of the marked effects as a cardiotonic agent in treating heart diseases, some species of the genus *Adonis* L. and their extracts have been widely used clinically in some countries, including the use of *A. vernalis* and *A. amurensis* in Russia and China. To provide a comprehensive review, the information on this genus was gathered via the internet and libraries, and the search languages were set to English and Chinese. The native languages of some articles (written in Bulgarian, Russian and German) as well as other factors including older publication dates and the absence of an English abstract made it impossible for us to cite and understand some articles. Although the pharmacological effects of this plant were widely studied in Russia before 1950s, much of the relevant literature is hard to access (Shikov et al., 2014). As a result, some older studies published in various languages were not included in this review and should be examined and reviewed further. Recently, the review of botany, traditional use, phytomedicine, pharmacology and toxicity of *A. vernalis* provides comprehensively information for this plant used in Europe (Latté, 2018).

According to the website www.theplantlist.org, 32 species from the genus were accepted as native to Europe and Asia. However, with the exception of *A. vernalis, A. aestivalis*, and *A. amurensis*, the phytochemistry and the modern pharmacology of most of the species have not been investigated comprehensively and clinically validated. Although *A. vernalis* has been become a well-known herbal medicine for cardioprotection, especially in Russia, Bulgaria, etc. (Popiliev et al., 1973; Sorokina, 1989; Wichtl, 1990), only small numbers of *in vitro* and *in vivo* studies on their cardioprotective effects are available (Popiliev et al., 1973). Considering that some clinical studies assayed about 50 years old are not valid anymore, the development of this genus should be paid more attention.

To date, more than 120 chemical components have been isolated and identified from the genus *Adonis*. With the exception of the cardiac glycosides, some well-known flavones in the genus also were isolated and identified with the wide pharmacological activities, including antioxidant, anti-microbial, anti-inflammatory, cardioprotective, neuroprotective, and anti-allergic properties, and these compounds should be paid more attention (George et al., 2017; Aziz et al., 2018; Guo et al., 2018; Kim et al., 2018).

Additionally, *A. vernalis* is a medicinal plant whose above-ground parts at the flowering or fruiting stages are harvested from the wild as a raw material for the pharmaceutical industry in China. In the past century, with the abundant use of *A. vernalis* as well as a lack of xerothermic habitats and slow plant growth among others, this resource has rapidly decreased and is close to extinction (Lange, 2000; Baier and Tischew, 2004; Denisow et al., 2014). Meanwhile, owing to the weak germination of the seeds and the slow growth intensity of the plants, the cultivation is unsuccessful (Galambosi, 1980a,b). Since 1982, it has been protected in several countries and the trade of this plant was banned in many East European countries (Lange, 2000). Therefore, investigation of sustainable usage practices is still necessary. This introduces the urgent problem of cultivation on a commercial scale, which would be useful for its conservation (Poluyanova and Lyubarskii, 2008).

In short, the phytochemical and pharmacological studies of the genus *Adonis* L. have received much interest. Extracts enriched in cardiac glycosides have been developed, and active compounds have been isolated and proven to provide cardioprotective activity. However, plants of this genus should be studied and developed further, with particular attention paid to conservation of resources and clinical testing.

## Author Contributions

XS and JZ conceived the review. XS, XG, XM, YZ, and BL wrote the manuscript. FY, HP, WW, and CW collected the literatures. YZ and CW edited the manuscript. All authors read and approved the final version of the manuscript.

## Conflict of Interest Statement

The authors declare that the research was conducted in the absence of any commercial or financial relationships that could be construed as a potential conflict of interest.

## References

[B1] Abduchamidov V. N. Hammermann A. Sokolov W. (1971). Adonis turkestanicus-eine neue aussichtsreiche Herz- und Gefasswirksame Heilpflanze. *Planta Med.* 20 272–277. 10.1055/s-0028-1099704 5166475

[B2] Aitova R. Z. Maslennikova V. A. Yamatova R. S. Gorovits M. B. Abubakirov N. K. (1971). Adonis glycosides III. Adonylic Acid. *Khim. Prir. Soedin.* 6 847–848.

[B3] Aziz N. Kim M. Y. Cho J. Y. (2018). Anti-inflammatory effects of luteolin: a review of *in vitro*, *in vivo*, and in silico studies. *J. Ethnopharmacol.* 225 342–358. 10.1016/j.jep.2018.05.019 29801717

[B4] Bae K. H. (2000). *The Medicinal Plants of Korea.* Seoul: Kyo-Hak PublishingCo, 128.

[B5] Bae K. H. You Y. J. Park J. Y. An R. B. Kim Y. H. Kang J. S. (2000). Screening of angiogenesis inhibitors from Korean plants (I). *Kor. J. Pharmacol.* 31 320–324.

[B6] Baek Y. S. Jung J. W. Lee S. H. Baek N. I. Park J. H. (2015). A new pregnane hexaglycoside from *Adonis multiflora*. *J. Korean Soc. Appl. Biol. Chem.* 58 895–899. 10.1007/s13765-015-0120-0

[B7] Baier A. Tischew S. (2004). Nature conservation management on dry grassland sites in Sachsen-Anhalt-Investigation on threatening factors and development strategies in the nature reserve “Lämmerberg und Vockenwinkel”. *Hercynia* 37 201–230.

[B8] Bekhterev V. M. (1898). The importance of a mixture of *Adonis vernalis* or digitalis with bromides or codeine in the treatment of epilepsy. *Rev. Psychiatry* 9:679.

[B9] Benson W. M. Edwards L. D. (1941). The utilization of pigeons for the biological assay of *Adonis vernalis*, N.F. VI. presented to the scientific section of the A. PH. A., detroit meeting. *J. Am. Pharm. Assoc.* 31 49–51. 10.1002/jps.3030310206

[B10] Büchner S. H. Kikuchi K. Chen K. K. (1965). A new glycoside of *Adonis vernalis*. *Life Sci.* 4 37–39. 10.1016/0024-3205(65)90029-9 14257062

[B11] Budzianowski J. Pakulski G. Robak J. (1991). Studies on antioxidative activity of come c-glycosyl flavones. *Pol. J. Pharmacol. Pharm.* 43 395–401. 1824129

[B12] Burrows G. E. Tyrl R. J. (2001). *Adonis L. In: Toxic Plants of North America.* Ames. IA: Iowa State University Press, 1006–1007.

[B13] Catalogue of Life (2017). *Adonis Flammea Jacq*. Available at: http://www.catalogueoflife.org/col/details/species/id/f572e94c982ad9cb96c6e67bdad5dc56. Retrieved 2017-04-21

[B14] Chen K. K. Anderson R. C. (1947). Digitalis-like action of some new glycosides and esters of strophanthidin. *J. Pharmacol. Exper. Therap.* 90 271–275. 20256486

[B15] Chen K. K. Brown Robbins E. Bliss C. I. (1942). The digitalis-like principles of calotropis compared with other cardiac substances. *J. Pharmacol. Exper. Therap.* 74 223–234.

[B16] Chen K. K. Hendernson F. G. Anderson R. C. (1951). Comparison of forty-two cardiac glycosides and aglycones. *J. Pharmacol. Exp. Ther.* 103 420–430. 14908859

[B17] Chernobai V. T. Komissarenko N. F. Litvinenko V. I. (1968). Structure of flavonoid glycoside from *Adnois vernalis*. *Khim. Prir. Soedin.* 4:51. 10.1007/s12272-012-0303-8 22477187

[B18] Chi L. G. Chen Y. Zhou M. Yu X. F. Chen Z. (1985). The cardiotonic effects of the total glycosides of *Adonis pseudoamurensis*. *Trad. Chin. Drug Res. Clin.* 1:214.

[B19] Chiang T. J. Mi C. S. (1958). A pharmacological study of FU Shou-Tsao herba *Adnonis amurensis*. *Acta Pharm. Sin.* 6 323–336.

[B20] Chinese Materia Editorial Committee and State Chinese Medicine Administration Bureau (2002). *Chinese Materia, Tibetan Volume.* Shanghai: Shanghai Scientific and Technical Publishers.

[B21] Committee for the Pharmacopoeia of P. R. China (1975). *Pharmacopoeia of P.R. China.* China: China Medical Science and Technology Press.

[B22] Coronary Disease Control Group of Liaoning TCM College’s Hospital (1971). The clinic observation of *Adonis amurensis* treating heart failure of 47 cases. *Liaoning. Med.* 5:44.

[B23] Dai Y. Zhang B. B. Xu Y. Liao Z. X. (2010). Chemical constituents of *Adonis coerulea* Maxim. *Nat. Prod. Res. Dev.* 22 594–596.

[B24] Das H. Raghav S. Gupta B. Das R. H. (2007). Anti-inflammatory compounds from medicinal plant *Ruta graveolens*. *Acta Horticult.* 756 389–398. 10.1007/s11626-014-9813-7 25274136

[B25] Davies R. L. Whyte P. B. (1989). *Adonis microcarpa* (pheasant’s eye) toxicity in pigs fed field pea screenings. *Aust. Vet. J.* 66 141–143. 10.1111/j.1751-0813.1989.tb09780.x 2735892

[B26] Degen A. V. (1932). Adonis-vergiftung. *Fortschr. Landwirtsch.* 7:556.

[B27] Deng S. X. Li C. D. He G. P. (1963). The cardiotonic effect of the total glycosides of *Adonis brevistyla* Franch. *Acta Pharm. Sin.* 10:677. 14097214

[B28] Denisow B. Wrzesie M. Cwener A. (2014). Pollination and floral biology of *Adonis vernalis* L. *(Ranunculaceae)* -a case study of threatened species. *Acta Soc. Bot. Pol.* 83 29–37. 10.5586/asbp.2014.001

[B29] Dong Y. (1981). The effect of the total cardiac glycosides treating premature ventricular contraction. *Fujian Med.* 1:48.

[B30] Drozd G. A. Koreshchuk K. E. Khapugina L. L. Miroshnikov E. V. (1971). Vitexin-a new flavone glycoside of *Adonis vernalis*. *Khim. Prir. Soedin.* 4 526–527. 10.1007/BF00564759 14402306

[B31] Egger K. (1965). Die ketocarotinoide in *Adonis annua* L. *Phytochemistry* 4 609–618. 10.1016/S0031-9422(00)86223-8

[B32] Egger K. Kleinig H. (1967a). Die ketocarotinoide in *Adonis annua* L.-II.: zur struktur der ester. *Phytochemistry* 6 437–440. 10.1016/S0031-9422(00)86302-5

[B33] Egger K. Kleinig H. (1967b). Die ketocartinoide in *Adonis annua* L.-III: vergleich mit synthetischen substanzen. *Phytochemistry* 6 903–905. 10.1016/S0031-9422(00)86040-9

[B34] Evdokimov P. K. (1979). Composition of *Adonis leiosepala*. *Khim. Prir. Soedin.* 5:736.

[B35] Felter H. W. Lloyd J. U. (2006). *Adonis-Pheasant’s Eye. King’s American Dispensatory.* Available at: http://www.ibiblio.org/herbmed/eclectic/kings/adonis.htmL

[B36] Flora of China (2018). Available at: http://www.efloras.org/florataxon.aspx?flora_id=2&taxon_id=100626

[B37] Franz G. (1971). Studies on the methylation of cymarose in *Adonis vernalis*. *Phytochemistry* 10 3001–3003. 10.1016/S0031-9422(00)97342-4

[B38] Galambosi B. (1980a). Results and cultivation of some wildflower medicinal plants in the “Szilasmenti” cooperative. *Acta Hort.* 96 343–352. 10.17660/ActaHortic.1980.96.37

[B39] Galambosi B. (1980b). Termesztési tapasztalatok magról vetett *Adonis vernalis* L. növényekkel. *Botanikai Közlemények.* 67 307–311. 28001500

[B40] Galey F. D. Holstege D. M. PlumLee K. H. Tor E. Johnson B. Anderson M. L. (1996). Diagnosis of oleander poisoning in livestock. *J. Vet. Diagn. Invest.* 8 358–364. 10.1177/104063879600800314 8844581

[B41] Genkina G. L. Éidler Y. I. Shakirov T. T. Yamatova R. S. (1972). Spectrophotometric determination of the cardenolides in the epigeal part of *Adonis chrysocyathus*. *Khim. Prir. Soedin.* 6 747–749. 10.1007/BF00564595

[B42] George V. C. Dellaire G. Rupasinghe H. P. V. (2017). Plant flavonoids in cancer chemoprevention: role in genome stability. *J. Nutrit. Biochem.* 45 1–14. 10.1016/j.jnutbio.2016.11.007 27951449

[B43] Ghorbani N. M. Azizian D. Sheidai M. Khatamsaz M. (2008). Pollen morphology of some *Adonis* L. species (Ranunculaceae) from Iran. *Iran. J. Bot.* 14 165–170.

[B44] Gostin I. N. (2011). Anatomical and micromorphological peculiarities of *Adonis vernalis* L. (Ranunculaceae). *Pak. J. Bot.* 43 811–820.

[B45] Greeff K. Kasperat H. (1961). Konvulsive und paralytische wirkungen von digitalis-glykosiden und geninen bei intracerebraler und intravenoser injektion an mausen (convulsive and paralytic effects of digitalis glycosides and genins after intracerebral and intravenous injection in mice). *Arzeimittelforschung* 11 908–909.

[B46] Gu P. K. Zhang Y. Chen Y. L. Shang M. Jin Z. J. (1981). The electrophysiology effect of Xinfugan on heart muscle cells. *J. Shanghai Sec. Med. Univ.* 2:10.

[B47] Gu X. L. Feng S. X. Ma B. R. Shi Y. (1989). Isolation and determination of some cardiac glycosides from *Adonis pseudoamurensis* by high-performance liquid chromatography. *Appl. Mod. Med.* 6 1–4.

[B48] Gu X. L. Ma B. R. Ren X. G. (1990). Separation and determination of relative content of cardiac glycosides from *Adonis amurensis* with high-performance liquid chromatography. *J. Notman Bethune Univ. Med. Sci.* 16 131–135.

[B49] Gu Z. L. Qian Z. N. Zang Y. Y. Chen B. Q. Wang Y. Q. (1980). The pharmacology of the total glycosides of *Adonis amurensis*. The effects of the total glycosides on the central nervous system of rabbits. *Zhong Cheng Yao Yan Jiu* 3 40–43.

[B50] Guo D. Hu X. Zhang H. Lu C. Cui G. Luo X. (2018). Orientin and neuropathic pain in rats with spinal nerve ligation. *Int. Immunopharmacol.* 58 72–79. 10.1016/j.intimp.2018.03.013 29558662

[B51] Heyl F. W. Hart M. C. Schmidt J. M. (1918). An examination of the leaves of *Adonis vernalis*. *J. Am. Chem. Soc.* 2 436–453. 10.1021/ja02235a018 13972598

[B52] Heyn C. C. Pazy B. (1989). The annual species of Adonis (Ranunculaceae)-A polyploid complexit. *Plant Sys. Evol.* 168 181–193. 10.1007/bf00936098

[B53] Hirsch H. Wagner V. Danihelka J. Ruprecht E. Sánchez-Gomez P. Seifert M. (2015). High genetic diversity declines towards the geographic range periphery of *Adonis vernalis*, a eurasian dry grassland plant. *Plant Biol.* 17 1233–1241. 10.1111/plb.12362 26122089

[B54] Hurst E. (1942). *Family Ranunculaceae. The Poison Plants of New South Wales.* Sydney: The Snelling Printing Works PTY. Ltd, 113–114.

[B55] Junior P. Wichtl M. (1980). 3-epi-periplogenin: ein neues cardenolid aus *Adonis vernalis*. *Phytochemistry* 19 2193–2197. 10.1016/S0031-9422(00)82222-0

[B56] Kamata T. Simpson K. (1987). Study of astaxthin diester extracted from *Adonis aestivealis*. *Comp. Biochem. Physiol.* 86B, 587–591.

[B57] Katz A. Reichstein T. (1947). Glykoside und aglykone; adonitoxin, das zweite stark herzwirksame Glykosid aus *Adonis vernalis*. *Pharm. Acta. Helv.* 22 437–459. 20264193

[B58] Keshan Research Group of Jilin Medical University First Clinical College Second Clinical College Third Clinical College of Jilin Medical University (1977). The clinical application of Binglianghua (*Adonis amurensis*). *Jilin Med. Univ.* 4 42–53.

[B59] Kim S. J. Pham T. H. Bak Y. Ryu H. W. Oh S. R. Yoon D. Y. (2018). Orientin inhibits invasion by suppressing MMP-9 and IL-8 expression via the PKC/ ERK/AP-1/STAT3-mediated signaling pathways in TPA-treated MCF-7 breast cancer cells. *Phytomedicine* 50 35–42. 10.1016/j.phymed.2018.09.172 30466990

[B60] Kloot P. M. (1976). The species of *Adonis* naturalized in Australia. *Muelleria* 3 300–207.

[B61] Komissarenko N. F. Korzennikova ÉP. Lushpa O. U. (1977). A chemical study of *Adonis tianschanicus*. *Khim. Prir. Soedin.* 2 287–288. 10.1007/BF00563973

[B62] Komissarenko N. F. Korzennikova P. Yatsyuk V. Ya (1973a). Cardenolides of *Adonis wolgensis*. *Khim. Prir. Soedin.* 6 806–807. 10.1007/BF00565712

[B63] Komissarenko N. F. Korzennikova P. Yatsyuk V. Ya (1973b). Cardenolides of *Adonis wolgensis*. *Khim. Prir. Soedin.* 9:433 10.1007/BF00565712

[B64] Komissarenko N. F. Yatsyuk Ya Korzennikova P. (1973c). Flavonoids of *Adonis wolgensis*. *Khim. Prir. Soedin.* 3:439 10.1007/BF00565720

[B65] Kooti W. Hasanzadeh-Noohi Z. Sharafi-Ahvazi N. Asadi-Samani M. Ashtary-Larky D. (2016). Phytochemistry, pharmacology, and therapeutic uses of black seed (*Nigella sativa*). *Chin. J. Nat. Med.* 14 0732–0745. 10.1016/S1875-5364(16)30088-7 28236403

[B66] Kooti W. Moradi M. Peyro K. Sharghi M. Alamiri F. Azami M. (2018). The effect of celery (*Apium graveolens* L.) on fertility: a systematic review. *J. Complement. Integ. Med.* 15:20160141. 10.1515/jcim-2016-0141 28985183

[B67] Kopp B. Krenn L. Kubelka E. Kubelka W. (1992). Cardenolides from *Adonis aestivalis*. *Phytochemistry* 31 3195–3198. 10.1016/0031-9422(92)83473-C 1368415

[B68] Kovaříková A. Chen K. K. (1965). Activities of newer glycosides of *Adonis vernalis* L. *Life Sci.* 4 41–43. 10.1016/0024-3205(65)90030-5 14257063

[B69] Kubo S. Kuroda M. Matsuo Y. Masatani D. Sakagami H. Mimaki Y. (2012). New cardenolides from the seeds of *Adonis aestivalis*. *Chem. Pharm. Bull.* 60 1275–1282. 10.1248/cpb.c12-00489 23036970

[B70] Kubo S. Kuroda M. Yokosuka A. Sakagami H. Mimaki Y. (2015). Amurensiosides L-P, five new cardenolide glycosides from the roots of *Adonis amurensis*. *Nat. Prod. Commun.* 10 27–32. 25920213

[B71] Kummer D. H. (1952). Vergiftungen bei pferden durch *Adonis* in luzerneheu (poisonings in horses with Adonis-contaminated alfalfa hay). *Tierarztl. Umsch.* 7 430–431.

[B72] Kuo C. H. Chang C. H. Sun C. H. Han H. Chin E. P. Chiang M. Y. (1962). A pharmacological study of *Adonis amurensis*. *Acta Pharm. Sin.* 9 135–144. 14073957

[B73] Kuroda M. Kubo S. Uchida S. Sakagami H. Mimaki Y. (2010). Amurensiosides A–K, 11 new pregnane glycosides from the roots of *Adonis amurensis*. *Steroids* 75 83–94. 10.1016/j.steroids.2009.10.008 19883671

[B74] Lamzhav A. (1975). *Untersuchungen Ueber das Vorkommen Von Herzwirksamen Glycosiden und Flavonoiden in Adonis Mongolica Sim.* Dissertation a. section biowissenschaften der karl marx universität. Leipzig.

[B75] Lamzhav A. (1983). Coumarins of *Adonis mongolica*. *Khim. Prir. Soedin.* 3:402.

[B76] Lange D. (2000). *Conservation and Sustainable use of Adonis vernalis, A Medicinal Plant in International Trade.* Rome: Food and Agriculture Organization of the United Nations.

[B77] Lateef T. Riaz A. Zehra A. Qureshi S. A. (2012). Antihyperlipidemic effect of *Adonis vernalis*. *J. Dow Univ. Health Sci.* 6 47–51.

[B78] Latté K. P. (2018). *Adonis vernalis* L. das frühlingsadonisröschen. *Z. Phytother.* 39 45–51. 10.1055/s-0044-100153

[B79] Lazareva D. N. (1975). Effect of methyluracil on the sensitivity of animals to cardiac glycosides and on the coronary blood flow. *Farmakol. Toksikol.* 38 311–313. 1241676

[B80] Lee J. H. Lee S. T. Seo Y. B. Yeo S. H. Lee N. S. (2003). A morphological reexamination on the genus *Adonis* L. sensu lato (Ranunculaceae) in Korea. *Korean J. Plant. Taxon.* 33 435–454. 10.11110/kjpt.2003.33.4.435

[B81] Lehmann H. D. (1984). Zur wirkung pflanzlicher glykoside auf widerstandsgefäße und kapazitätsgefäße. *Arzneimittelforschung* 34 423–429. 6540100

[B82] Lenel-Pekelis (1949). Bio-assay of *Adonis vernalis* glycosides: mercier, f: *cardiologia*. *Am. Heart J.* 37:314 10.1016/0002-8703(49)90602-X

[B83] Liu J. Cui X. W. (2007). Determination of convallatoxin from *Adonis amurensis* by high-performance liquid chromatography. *Chin. Trad. Herb. Drug* 38 617–618.

[B84] Ma B. R. Zhao Q. C. Yang S. J. He L. (1985). A preliminary study on the chemical components of *Adonis pseudoamurensis* W.T.Wang. *J. Norman Bethune Health Sci. Univ.* 11:371.

[B85] Maham M. Sarrafzadeh-Rezaei F. (2014). Cardiovascular effects of *Adonis aestivalis* in anesthetized sheep. *Vet. Res. Forum.* 5 193–199. 25568718 PMC4279646

[B86] Maiden J. H. (1912). A new poison plant (*Adonis autumnalis*). *Agri. Gaz. New South Wales* 23:810. 29753868

[B87] Mathe A. Mathe J. I. (1979a). Data to the cardiac glycoside content of *Adonis vernalis* L. *in Hungary*. *Herb. Hung.* 18 115–124.

[B88] Mathe A. Mathe J. I. (1979b). Preliminary survey of the variability of the cardiac glycoside production of *Adonis vernalis* L. native in Hungary. *Herb. Hung.* 18 21–28.

[B89] Matthiesen U. Pauli G. F. Junior P. (1992). Aleppotrioloside, an aliphatic alcohol glycoside from *Adonis aleppica*. *Phytochemistry* 31 2522–2524. 10.1016/0031-9422(92)83314-O

[B90] May G. Willuhn G. (1978). Antiviral activity of aqueous extracts from medicinal plants in tissue cultures. *Arzneimittel Forschung* 28 1–7. 204315

[B91] Mohadjerani M. Tavakoli R. Hosseinzadeh R. (2014). Fatty acid composition, antioxidant and antibacterial activities of *Adonis wolgensis* L. extract. *Avicenna J. Phytomed.* 4 24–30. 25050298 PMC4103726

[B92] Munch J. C. Jr Krantz J. C. (1934). Pharmacological and chemical studies of the digitalis group. *I.* Adonis, apocynum and convallaria. *J. Am. Pharm. Assoc.* XXIII:988.

[B93] Nosal M. A. Nosal I. M. (1960). *Lekarstvennuiye Rasteniyai Sposobuiikh Primrniyav Narodye (Medicinal Plants and the Ways that they are used by people).* Kievp: State medical publishing, 256.

[B94] Orhan I. E. Gokbulut A. Senol F. S. (2017). Adonis, sp., *Convallaria sp*., Strophanthus sp., Thevetia sp., and Leonurus sp. -cardiotonic plants with known traditional use and a few preclinical and clinical studies. *Curr. Pharm. Design.* 23 1051–1059. 10.2174/1381612822666161010104548 27748195

[B95] Pauli G. F. (1995). Adoligoses, oligosaccharides of rare sugars from *Adonis aleppica*. *J. Nat. Prod.* 58 483–494. 10.1021/np50118a002 7623026

[B96] Pauli G. F. Junior P. (1993). Alepposides, cardenolide oligoglycosides from *Adonis aleppica*. *J. Nat. Prod.* 56 67–75. 10.1021/np50091a010 8450322

[B97] Peter J. Max W. (1980). *3-epi*-periplogenin: ein neues cardenolid aus *Adonis vernalis*. *Phytochem* 19 2193–2197. 10.1016/S0031-9422(00)82222-0

[B98] Pitra J. Čekan Z. (1961). Herzwirksame glykoside III. Cardenolide des adonisröschens (*Adonis vernalis* L.). *Coll. Czech. Chem. Commun.* 26 1551–1558. 10.1135/cccc19611551

[B99] Poláková A. Čekan Z. (1965). Isolation and structure of cardenolides from *Adonis vernalis*. *Cesk. Farm.* 14 307–315. 5892444

[B100] Poluyanova V. I. Lyubarskii E. L. (2008). On the ecology of seed germination in *Adonis vernalis*. *Russian J. Eco.* 39 68–69. 10.1134/S1067413608010116

[B101] Ponomarenko A. A. Komissarenko N. F. Stukkei K. L. (1971a). Cardenolides from *Adonis amurensis*. *Khim. Prir. Soedin.* 7 848–849.

[B102] Ponomarenko A. A. Komissarenko N. F. Stukkei K. L. (1971b). Coumarins from *Adonis amurensis*. *Khim. Prir. Soedin.* 5 661–662. 19023537

[B103] Popiliev I. Belovejdov N. Gelinov H. (1973). Clinical therapeutic studies with the cardiotonic preparation AV 2 (*Adonis vernalis* glycosides). *Savremenna Med.* 24 29–31.

[B104] Qin Y. (2000). Ethanol extract of *Adonis amurensis* Regel et Radde‘s influence on the rabbit‘s movement of atrial muscle *in vitro*. *J. Tonghua Teach. Coll.* 5:49.

[B105] Renstrøm B. Berger H. Liaaen-Jensen S. (1981). Esterified, optical pure (3S, 3‘S)-astaxanthin from flowers of *Adonis annua*. *Biochem. Sys. Eco.* 9 249–250. 10.1016/0305-1978(81)90003-X

[B106] Sato Y. Hirano M. Nitta I. Azuma J. Hayashi K. Mitsuhashi H. (1971). Components of adonis plants. *Chem. Pharm. Bull.* 19 202–205. 10.1248/cpb.19.202

[B107] Shang X. F. Guo X. Yang F. Li B. Pan H. Miao X. L. (2017). The toxicity and the acaricidal mechanism against *Psoroptes cuniculi* of the methanol extract of *Adonis coerulea* Maxim. *Vet. Parasitol.* 240 17–23. 10.1016/j.vetpar.2017.04.019 28576339

[B108] Shang X. F. Miao X. L. Wang D. S. Li J. X. Wang X. Z. Yan Z. T. (2013). Acaricidal activity of extracts from *Adonis coerulea* maxim. against *Psoroptes cuniculi in vitro and in vivo*. *Vet. Parasitol.* 195 136–141. 10.1016/j.vetpar.2012.12.057 23352106

[B109] Shang X. F. Tao C. X. Miao X. L. Wang D. S. Tangmuke Dawa (2012). Ethno-veterinary survey of medicinal plants in Ruoergai region. Sichuan province, China. *J. Ethnopharmacol.* 142 390–400. 10.1016/j.jep.2012.05.006 22634202

[B110] Shen X. T. Qian Y. X. Wang S. B. Lin J. B. Ding J. M. Yang Z. C. (1983). Action of k-strophanthin or Adonside with antiarrhythmic drugs on aconitine induced cardiac arrhythmia in mice. *Acta Acad. Med. Primae Shanghai.* 10 41–46.

[B111] Shi L. Wang D. S. Gu X. H. Pan J. X. (1979). The pharmacology of the total glycosides of *Adonis amurensis*. I The effects of the total glycosides on the functions of the left ventricle and K+ metabolism of myocardium. *ZhongChengYao YanJiu* 4 27–32.

[B112] Shikov A. N. Pozharitskaya O. N. Makarov V. G. Wagner H. Verpoorte R. Heinrich M. (2014). Medicinal plants of the Russian pharmacopoeia; their history and applications. *J. Ethnopharmacol.* 154 481–536. 10.1016/j.jep.2014.04.007 24742754

[B113] Shimizu Y. Sato Y. Mitsuhashi H. (1967). Isolation and structure of adonilide. *Chem. Pharm. Bull.* 15 2005–2006. 5590709

[B114] Shimizu Y. Sato Y. Mitsuhashi H. (1969a). Isolation and characterization of fukujusonorone, an 18-norpregnane derivative from *Adonis amurensis* Regel et Radd. *Experientia* 25 1129–1130. 10.1007/BF01900226 5357805

[B115] Shimizu Y. Sato Y. Mitsuhashi H. (1969b). Isolation and structures of new pregnane derivatives from *Adonis amurensis*. *Chem. Pharm. Bull.* 17 2391–2394. 10.1248/cpb.17.2391 5391753

[B116] Shimizu Y. Sato Y. Mitsuhashi H. (1978). A study of the chemical constituents of *Adonis amurensis*. *Lloydia* 41 1–16.

[B117] Shuguang Medical Team of Anshan City Department of Pharmacy of Anshan Steel Hospital and Department of Pharmacy of Anshan Medical School (1973). The electrocardiogram of cats treated by the total glycosides of *Adonis amurensis*. *Xin Yiyao Xue Zazhi* 10:24.

[B118] Sokolov Ya (2000). *Phytotherapy and Phytopharmacology: The Manual for Doctors.* Moscow: Medical News Agency.

[B119] Sorokina A. A. (1989). Spring adonis (*Adonis vernalis* L.). *Med. Sestra* 48 43–45. 2593790

[B120] Sun W. (1988). The poisoning case induced by *Adonis amurensis*. *Chin. J. Hospit. Pharm.* 8:38.

[B121] The plant list (2013). Available at: http://www.theplantlist.org/1.1/browse/A/Ranunculaceae/Adonis/

[B122] Thieme H. Lamzhav A. (1976). Ueber die cardenolidglycoside von *Adonis mongolica* Sim. *Pharmazie* 25:1976. 996079

[B123] Turova A. D. Sapozhnikova E. N. (1989). *Medicinal Plants of USSR and their applications.* Moscow: WHO.

[B124] Utkin L. A. (1931). Traditional medicinal plants of siberia. *Proc. Sci. Res. Institutes Ind.* 434:24.

[B125] Vogel V. G. Kluge E. (1961). Comparative studies on the diuretic action of some steroids with cardiac action. *Arzneimittelforschung* 11 848–850. 13926417

[B126] Wagler M. (2001). The homeopathic pharmacopoeia 2001: new regulations for homeopathic drugs. *Deutsche Apothr. Zeit.* 141 86–89.

[B127] Wagner H. Rosprim L. Galle K. (1975). Endgültige struktur von adonivernith aus *Adonis vernalis*. *Phytochemistry* 14 1089–1091. 10.1016/0031-9422(75)85193-4

[B128] Wang D. Liu L. J. Liu M. Li R. J. Liu M. Y. (1991). The change regulation of total cardiac glycoside at the different phase of *Adonis amurensis* Regel et Radde. *Chin. Trad. Herb. Drugs* 14:7.

[B129] Wang D. S. Zhou Z. Q. Wang S. L. Wang X. Y. Gu X. T. (1981). The pharmacology of the total glycosides of *Adonis amurensis*. III The diuretic effects of the total glycosides and the relationship between the effects and ion in urine. *ZhongChengYao Yanjiu* 5 35–36.

[B130] Wang M. X. Feng W. X. (1982). The abnormal of heart rate induced by *Adonis amurensis*. *Chin. J. Pract. Intern. Med.* 2:50.

[B131] Wichtl M. (1990). Herbal medicines in cardiovascular disorders. *Deutsche Apoth. Zeit* 130 1251–1256.

[B132] Wichtl M. Jentzsch K. Türk E. (1972). Strophantidine fucoside, a new cardenolide glycoside from *Adonis vernalis* L. *Monatsh. Chem.* 103 889–895. 10.1007/BF00905451

[B133] Winkler C. Wichtl M. (1985). New cardiac glycosides from *Adonis vernalis*. *Pharm. Acta Helv.* 60 243–247.

[B134] Winkler C. Wichtl M. (1986). Neue cardenolide aus *Adonis vernalis*. *Planta Med.* 52:68 10.1055/s-2007-969076

[B135] Woods L. W. Filigenzi M. S. Booth M. C. Rodger L. D. Amold J. S. Puschner B. (2004). Summer pheasant’s eye (*Adonis aestivalis*) poisoning in three horses. *Vet. Pathol.* 41 215–220. 10.1354/vp.41-3-215 15133169

[B136] Woods L. W. George L. W. Anderson M. L. Woods D. M. Filigenzi M. S. Puschner B. (2007). Evaluation of the toxicity of *Adonis aestivalis* in calves. *J. Vet. Diagn. Invest.* 19 581–585. 10.1177/104063870701900523 17823409

[B137] Woods L. W. Puschner B. Filigenzi M. S. Woods D. M. George L. W. (2011). Evaluation of the toxicity of *Adonis aestivalis* in sheep. *Vet. Rec.* 168:49. 10.1136/vr.c6231 21257561

[B138] Yang W. H. Zhang X. W. Xu W. J. Huang H. Y. Ma Y. Bai H. (2015). Overview of pharmacological research on *Adonis L*. *Agri. Sci. Tech.* 16 626–628.

[B139] Yatsyuk V. Y. Dolya V. S. Gella V. (1983). A phytochemical investigation of the epigeal part of *Adonis aestivalis*. *Khim. Prir. Soedin.* 5:641.

[B140] Yatsyuk V. Y. Komissarenko N. F. Gella ÉV. (1976). Cardenoloids of *Adonis wolgensis*. *Khim. Prir. Soedin.* 5:672 10.1007/BF00565218

[B141] Yin L. Zhang Y. Tian H. Y. Jiang R. W. (2014). Chemical constituents from *Adonis amurensis*. *Chin. Trad. Herb. Drugs* 45:3361.

[B142] You Y.-J. Yong K. Nguyen-Hai N. Byung-Zun A. (2003). Inhibitory effect of *Adonis amurensis* components on tube-like formation of human umbilical venous cells. *Phytother. Res.* 17 568–570. 10.1002/ptr.1184 12749003

[B143] Zhang H. D. Zhang S. J. Chen Y. Z. (1991). Studies on chemical constituents of *Adonis coerulea* maxim-a tibetan medicinal herb. *J. Lanzhou Univ.* 27 88–92.

[B144] Zhang L. H. Peng Y. J. Xu X. D. Wang S. N. Yu L. M. Hong Y. M. (2015). Determination of other related carotenoids substances in astaxanthin crystals extracted from *Adonis amurensis*. *J. Oleo Sci.* 64 751–759. 10.5650/jos.ess14203 26062642

[B145] Zhang X. E. (1999). Two cases of abnormal heart rate induced by *Adonis amurensis*. *J. Electrocard.* 18:53.

[B146] Zheng H. C. (1975). Translated from makciotoba C., 1975. *Pactè Pecyp* 11:512.

